# Host–Guest Interactions of Cucurbit[7]uril with Nabumetone and Naproxen: Spectroscopic, Calorimetric, and DFT Studies in Aqueous Solution

**DOI:** 10.3390/molecules30122558

**Published:** 2025-06-12

**Authors:** David Klarić, Valentina Borko, Jelena Parlov Vuković, Viktor Pilepić, Ana Budimir, Nives Galić

**Affiliations:** 1 Department of Chemistry, Faculty of Science, University of Zagreb, Horvatovac 102a, 10000 Zagreb, Croatia; dklaric@chem.pmf.hr (D.K.); ngalic@chem.pmf.hr (N.G.); 2 Faculty of Pharmacy and Biochemistry, University of Zagreb, A. Kovačića 1, 10000 Zagreb, Croatia; valentina.borko@pharma.unizg.hr (V.B.); viktor.pilepic@pharma.unizg.hr (V.P.); 3 Ruđer Bošković Institute, NMR Centre, Bijenička cesta 54, 10000 Zagreb, Croatia; jparlovv@irb.hr

**Keywords:** nabumetone, naproxen, cucurbit[7]uril, inclusion complex, ITC, HR-MS, NMR, DFT, IGMH

## Abstract

The complexation of nabumetone (NAB) and naproxen (NAP) with cucurbit[7]uril (CB7) was investigated in aqueous solution by isothermal titration microcalorimetry, mass spectrometry, NMR spectroscopy, and computation methods. High-resolution mass spectrometry was used for the determination of the binding stoichiometry and the gas-phase stability of the drug–CB7 complex. The doubly charged NH_4_^+^ or Na^+^ adducts of the 1:1 complex were observed in the mass spectra. The dissociation of complexes was monitored at different collision energies, (1–16) eV, leading to the neutral loss of NH_3_ and the drug, with charge retention observed on CB7. By performing ITC experiments, all the thermodynamic parameters were determined for the NAB-CB7 complex in water at 25 °C. The corresponding values amounted to the following: log*K* = 4.66 ± 0.01; Δ_r_*G*° = −26.7 ± 0.1 kJ/mol; Δ_r_*H*° = −20.2 ± 0.7 kJ/mol; *T*Δ_r_*S*° = 6.4 ± 0.8 kJ/mol, i.e., the formation of the inclusion complex is enthalpy driven and has a favorable entropy. The inclusion phenomena were further confirmed by NMR spectroscopy (^1^H, ROESY, and DOSY), suggesting the encapsulation of the naphthalene ring of both drugs inside the CB7 cavity. The results of the DFT calculations and the IGMH analysis were in accordance with the experimental ones, suggesting that van der Waals interactions play a major role in drug–CB7 complexation.

## 1. Introduction

The formation of host–guest complexes by the incorporation of a small organic molecule into the cavity of a macrocyclic molecule is a very attractive field of research in supramolecular chemistry, which has developed enormously in recent decades [1]. The supramolecular, reversible, noncovalent host–guest interaction is a simple and practical method to modulate the physicochemical properties of the guest molecule. The modulation of physicochemical properties through the formation of the complex is particularly important in pharmaceutical chemistry, where the modulation of drug solubility, stability, and bioavailability is of great importance [2]. Among the macrocyclic host molecules, such as cyclodextrins, calixarenes and pillarenes, cucurbiturils have emerged as a particularly interesting class due to their exceptional complexation properties and biocompatibility [3,4,5].

**Figure 1:**
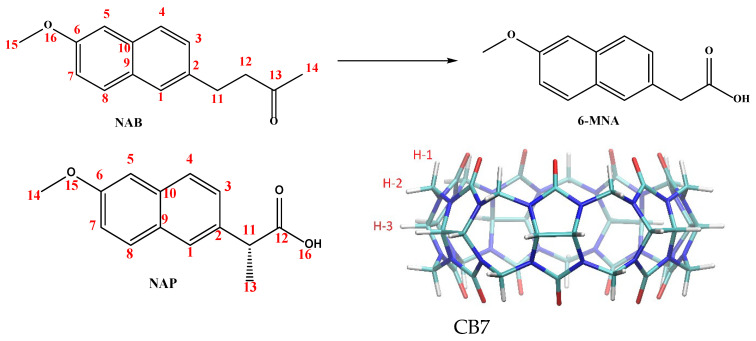
Molecular structure of nabumetone (NAB), its active metabolite (6-MNA), naproxen (NAP), and cucurbit[7]uril.

Cucurbit[n]urils are a family of macrocyclic compounds characterized by pumpkin-shaped ring structures composed of n (n = 5, 6, 7, 8, and 10, abbreviated as CBn) glycoluril monomer units linked by a pair of methylene groups [3,4,5,6]. The cyclic structure thus creates two identical, partially negatively charged hydrophilic carbonyl portals on each side and a hydrophobic cavity whose size increases with the number of glycoluril units. Due to these properties, CBs can form stable and reversible host–guest complexes with various cationic or neutral guest molecules via noncovalent interactions, such as ion–dipole interactions, hydrogen bonding, and hydrophobic effects. In particular, CB7 (Figure 1), with good water solubility and a suitable cavity size (5.4 Å portal diameter, 73 Å cavity diameter, and 279 Å^3^ cavity volume) to include various aromatic molecules, has considerable potential for biomedical applications [7,8,9,10]. Indeed, there are many examples of drugs or biologically active compounds that have been studied in combination with CB7, and they cover a wide range of applications, e.g., local anesthesia [11], the treatment of infections [12,13], anticancer drugs [14], etc. By forming host–guest complexes, CB7 can regulate drug release and alleviate side effects, improving the stability and water solubility of drugs [15]. For example, the cytotoxicity of heptaplatin, a platinum chemotherapeutic agent for the treatment of colorectal tumors, to the normal colorectal cell can be significantly reduced by the formation of host−guest complexes with heptaplatin-CB7, thereby reducing the cytotoxicity of heptaplatin during administration. More significantly, the antitumor activity of the heptaplatin-CB7 complex is higher than that of heptaplatin [16]. Compared to β-CD-based formulations, CB7 exhibits higher binding constants with the guest, making it more efficient than β-cyclodextrin (β-CD) as an excipient to improve the solubility of poorly soluble drugs. Recently, the stability constant of the CB7 complex with the nonsteroidal anti-inflammatory drug (NSAID) piroxicam was reported to be about 70-fold higher than that with β-CD. In addition, the CB7–piroxicam inclusion complex showed a rapid dissolution rate in the gastric environment and a significantly higher oral bioavailability compared to the complex with β-CD, resulting in an improved anti-inflammatory effect in both mouse and rat models [17].

NSAIDs are widely used in clinical practice for the treatment of pain, inflammation, and fever. They also serve as valuable molecular systems in host–guest chemistry due to their diverse structures, amphiphilic character, and biologically relevant functionalities. In this study, we selected two structurally similar NSAIDs as guest molecules to investigate the host–guest interactions with CB7. The first molecule used as a guest, naproxen (NAP, Figure 1), is a long-known NSAID that is active in its administered form. The second, nabumetone (NAB, Figure 1), is a prodrug that generates the active molecule 6-methoxy-2-naphthylacetic acid (6-MNA, Figure 1) through metabolic reactions. Both drugs have been on the market for a long time but are still being researched today [18,19,20].

Naproxen, ((S)-2-(6-methoxynaphthalen-2-yl) propanoic acid) is a widely prescribed NSAID used in the management of musculoskeletal pain, osteoarthritis, rheumatoid arthritis, and dysmenorrhea [21]. Its mechanism of action involves the non-selective inhibition of cyclooxygenase (COX-1 and COX-2), which reduces the synthesis of the prostaglandins responsible for pain and inflammation. Structurally, naproxen contains a rigid naphthalene ring, a methoxy substituent, and a chiral α-methylacetic acid side chain. At a physiological pH, this molecule is predominantly present in its anionic form (p*K*_a_ ≈ 4.2) [22] and has both hydrophobic (aromatic) and polar (carboxylate) domains.

Nabumetone (4-(6-methoxynaphthalen-2-yl) butan-2-one, Figure 1), on the other hand, is a non-acidic prodrug that is clinically used to relieve pain and inflammation in the treatment of patients with osteoarthritis or rheumatoid arthritis [23]. Structurally, nabumetone shares the methoxy-substituted naphthalene ring with naproxen but has a neutral butanone side chain, resulting in a non-ionizable, more lipophilic profile.

Given the different charge states, hydrophobicities (Log*P*_NAB_ ≈ 3.22 vs. Log*P*_NAP_ ≈ 2.99) and functional groups of naproxen and nabumetone (carboxylic acid vs. ketone), they are an ideal model pair to study how structural variations and acid–base properties affect complexation behavior with CB7. The binding behavior between CB7 and these two NSAIDs was investigated by isothermal titration microcalorimetry, high-resolution mass spectrometry, ^1^H NMR spectroscopy, DFT calculations, and IGMH. One previous study reported a CB7 inclusion complex of naproxen with a stability constant determined by ^1^H NMR spectroscopy [24]. However, to the best of our knowledge, this is the first study to report the formation of a stable inclusion complex between NAB and CB7 in aqueous solution. The binding constant and thermodynamic parameters were determined using isothermal titration microcalorimetry. High-resolution mass spectrometry was used to determine the binding stoichiometry of the NAP-CB7 and NAB-CB7 complexes. In addition, the inclusion phenomena were further investigated by NMR spectroscopy (^1^H, ROESY and DOSY). The DFT calculations and IGMH analysis were applied to determine the structure and intermolecular bonding interactions of the NAP-CB7- and NAB-CB7 host–guest systems. Our findings provide insights into the design of CB7-based drug delivery systems and advance the understanding of host–guest interactions involving pharmaceutically relevant molecules with differing functional characteristics.

## 2. Results and Discussion

### 2.1. Mass Spectrometry

**Figure 2:**
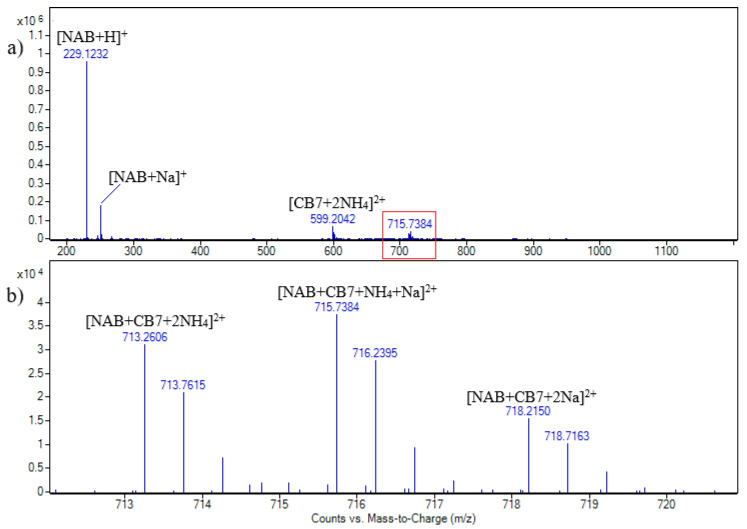
ESI + HRMS spectrum of nabumetone and CB7 solution in molar ratio 1:1. c (NAB) = 2.15 × 10^−5^ M (**a**); enhanced view of ion signals between *m/z* 712 and 720 (**b**).

The application of soft ionization methods, such as electrospray ionization (ESI) and matrix-assisted laser desorption ionization (MALDI), makes mass spectrometry a useful tool in the study of noncovalent complexes of cucurbiturils [25,26]. By conducting collision-induced dissociation (CID) experiments, the differentiation between externally bound complexes from inclusion complexes can be obtained [27]. Detailed mass spectrometric analyses of NAB-CB7 and NAP-CB7 systems were performed. The MS spectrum of a CB7 solution in the ESI+ mode of ionization is shown in Figure S1. The signal at *m/z* 599.2 was assigned to [CB7 + 2NH_4_]^2+^, and the signals at 601.7 and 604.1 were assigned to the [CB7 + NH_4_ + Na]^2+^ and [CB7 + 2Na]^2+^ doubly charged ions, respectively. The difference between the theoretical and the measured masses was less than 5 ppm in all cases. By applying the CE of 20 eV, the complete loss of NH_3_ from the [CB7 + 2NH_4_]^2+^ ion was not observed, indicating strong binding (Figure S2, Table S1). The MS spectrum obtained in ESI–mode of ionization (Figure S3) is characterized by the signal at *m/z* 1207.3, which was assigned to the [CB7 + HCOO]^−^ ion. The MS spectra of the NAB-CB7 solution are shown in Figure 2, and the assignation of all the signals is shown in Table S2.

As can be seen, the signals assigned to the [CB7 + NAB + 2NH_4_]^2+^, [CB7 + NAB + NH_4_ + Na]^2+^, and [CB7 + NAB + 2Na]^2+^ doubly charged ions are at *m/z* values of 713.3, 715.7, and 718.2, respectively. The cucurbituril adducts with ammonia or alkali metal ions are usually observed in MS spectra, acquired both in positive and negative ion modes. The signals at *m/z* 229 and 251 (Figure 2) are assigned to the [NAB + H]^+^ and [NAB + Na]^+^ singly charged ions, respectively [19,20].

**Figure 3:**
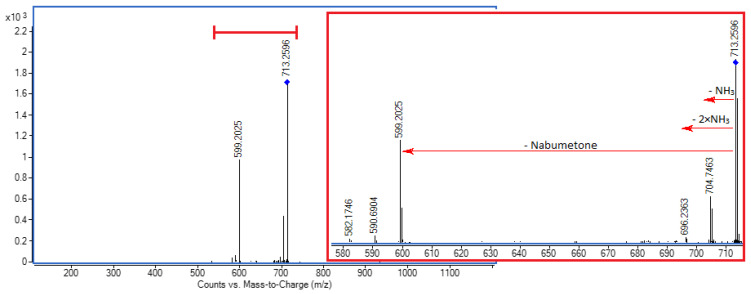
MS/MS spectrum of [CB7 + NAB + 2NH_4_]^2+^ ion (*m/z* 713.2606) at 5 eV; inset showing enlarged spectral region between *m/z* 580 and 715.

The results of the MS/MS experiments are shown in Figure 3 and Table S3. As expected, the doubly charged ions need less energy for fragmentation, so the loss of the NH_3_ units and the NAB molecule is evident, even at the CE of 5 eV. The remaining MS/MS spectra of the [CB7 + NAB + 2NH_4_]^2+^ and [CB7 + NAB + NH_4_ + Na]^2+^ ions acquired at 5, 10, and 15 or 20 eV can be found in the SI (Figures S4–S8).

**Figure 4:**
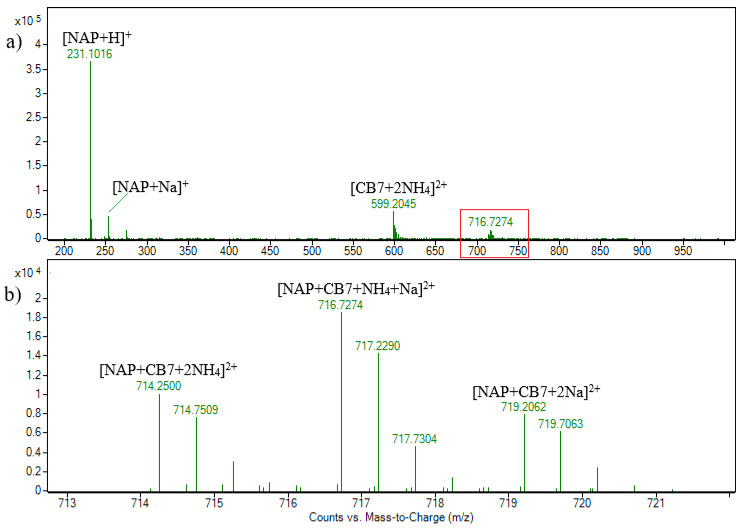
ESI + HRMS spectrum of naproxen and cucurbituril7 solution in molar ratio 1:1. c(NAP) = 2.15 × 10^−5^ M (**a**); enhanced view of ion signals between *m/z* 713 and 722 (**b**).

The MS spectra of the NAP-CB7 solution in ESI+ mode are shown in Figure 4, and the assignation of all the signals is shown in Table S4. The observed MS signals align fully with those previously explained for the NAB-CB7 system, indicating identical behavior under similar conditions. The MS/MS spectra obtained for the [CB7 + NAP + 2NH_4_]^2+^ signals (Figures S9–S11) also correspond closely to those observed in the previously explained case of [CB7 + NAB + 2NH_4_]^2+^, further supporting the consistency of the fragmentation patterns, where precursor fragmentation yields a predominant product ion with the *m/z* value of 599.2, which corresponds to [CB7 + 2NH_4_]^2+^. However, some differences were noticed. The signal with the *m/z* value of 682.7 corresponds to the loss of a single NH_3_ unit and an additional neutral loss of an HCOOH molecule from the NAP.

Unlike the NAB-CB7, the MS spectra of the NAP-CB7 solution in ESI–mode (Figure S12) was characterized by a signal at *m*/*z* 1391.4, which was assigned to the [CB7 + NAP − H]^−^ ion. All these results suggest that the 1:1 NAB-CB7 and NAP-CB7 inclusion complexes are formed in the solution and are fragmented by the disruption of the noncovalent interaction during CID experiments in the gas phase.

The gas-phase stabilities of weakly bound noncovalent complexes were recently investigated and compared using CID experiments and by determining CE_50_ values for the precursor ions [28,29]. To minimize charge-related effects and better approximate solution-phase stability, such experiments are ideally conducted on protonated and deprotonated species. However, due to the poor ionization of the protonated (and deprotonated, in the case of NAB) NAB-CB7 and NAP-CB7 complexes, CID experiments were instead performed on doubly charged ammonium adduct ions in both cases. While previous studies by Zhang et al. have shown that CB7 exhibits a higher binding affinity toward NH_4_⁺ and Na⁺ compared to H⁺ in a solution [30], our gas-phase results similarly suggest a preferential stabilization of alkali and ammonium cations over protons, as no protonated CB7 species were detected. Notably, protonated species were not detected, even when 0.1% formic acid was used as the sample diluent.

**Figure 5:**
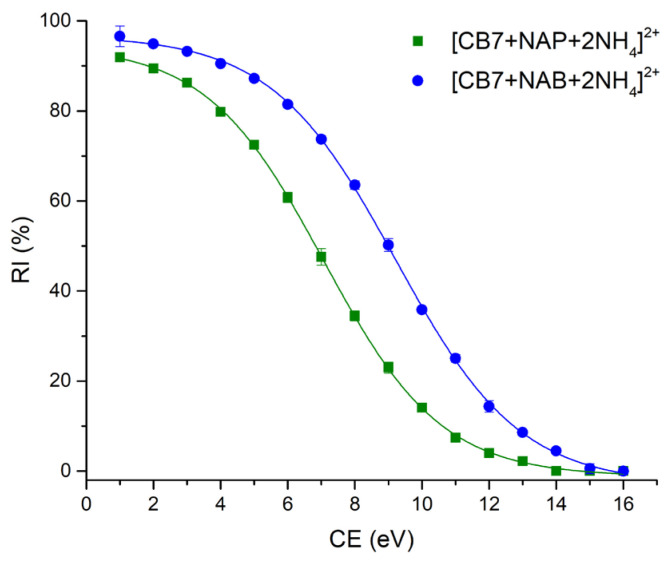
The relative intensities of complex molecular ions [CB7 + NAP + 2NH_4_]^2+^ (■) and [CB7 + NAB + 2NH_4_]^2+^ (●) as a function of the collision energy (CE). Error bars represent the standard deviation for the quadriplicate analyses.

The precursor ions of the NAP-CB7 and NAB-CB7 complexes were completely depleted at CE values of 14 and 15 eV, respectively. The corresponding breakdown curves are illustrated in Figure 5.

The calculated CE_50_ values for the depletion of [CB7 + NAP + 2NH_4_]^2+^ and [CB7 + NAB + 2NH_4_]^2+^ were 7.07 ± 0.02 (R^2^ = 0.99) and 9.22 ± 0.04 (R^2^ = 0.99), respectively, indicating that the NAB-CB7 complex exhibits greater gas-phase stability compared to the structurally similar NAP-CB7 noncovalent complex.

### 2.2. NMR Spectroscopy

NMR spectroscopy is a powerful technique for elucidating the structural properties of inclusion drug–CB7 complexes in solution. CB7 protons are pointing away from the cavity, so the change in the chemical shift of these protons is usually not observed upon complexation [31]. However, upon inclusion, the noticeable shift of drug protons occurs, so the upfield shift (lower ppm) due to shielding is a confirmation of the inclusion phenomena [31]. On the other hand, the drug protons located outside, but in the vicinity of CB7 portals, are deshielded, and the corresponding peaks are slightly shifted downfield. To gain insights into the structure of the NAB-CB7 and NAP-CB7 complexes, an NMR study was carried out.

**Figure 6:**
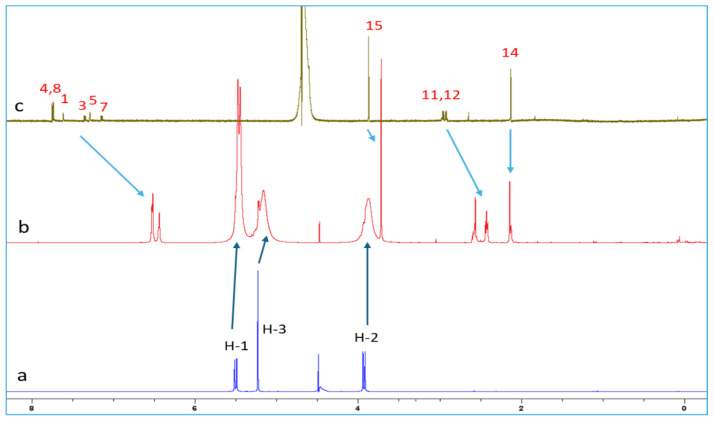
^1^H NMR spectra of (**a**) CB7; (**b**) NAB-CB7 1:1 complex; and (**c**) NAB in D_2_O at 25 °C.

The proton chemical shifts of NAB-CB7 were assigned using the ^1^H-NMR technique and previously published research data [19,32,33]. Due to the low solubility of NAB and CB7 in water, only the ^1^H NMR spectra with the water suppression module were recorded for these molecules. A comparison of the spectrum of NAB (Figure 6c) with the proton spectrum of the NAB-CB7 1:1 complex (Figure 6b) shows that the solubility of the complexed NAB is much higher than that of the free form. An analysis of the ^1^H NMR spectra of NAB, CB7, and the NAB-CB7 complex showed a significant change in the chemical shift of NAB after complex formation, especially the aromatic NAB protons (Figure 6, Table 1). The upfield shift of these protons, as well as protons 11 and 12, suggested the inclusion of the whole NAB molecule inside the cavity, while the methyl groups (protons 14 and 15) are exposed to the bulk near the CB7 portals. These results are similar to those previously reported for the complexation of NAB with β–CDs [19].

**Figure 7:**
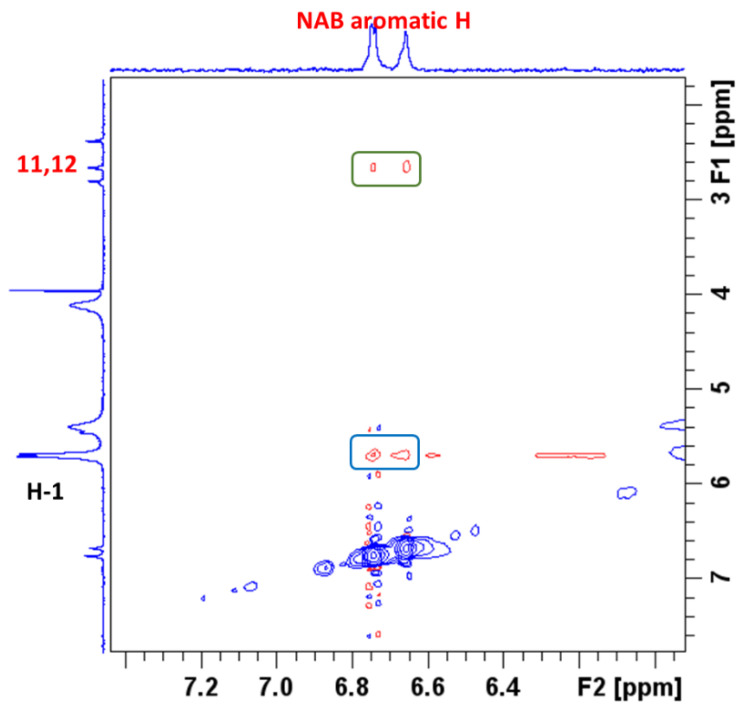
A part of the ROESY spectrum, showing the intermolecular interactions of NAB with CB7 in a 1:1 complex in D_2_O at 25 °C.

To better understand the intermolecular interactions between NAB and CB7 molecules in the complex, 2D ROESY experiments were performed. ROESY crosspeaks were assigned between the aromatic protons of NAB and the H-1 protons of CB7 (Figure 7), confirming the proximity between the two structural parts. Figure 7 shows a part of the NAB-CB7 spectrum, which shows both intermolecular (marked in blue) and intramolecular (marked in green) ROE contacts. The complete ROESY spectrum is shown in Figure S13.

**Figure 8:**
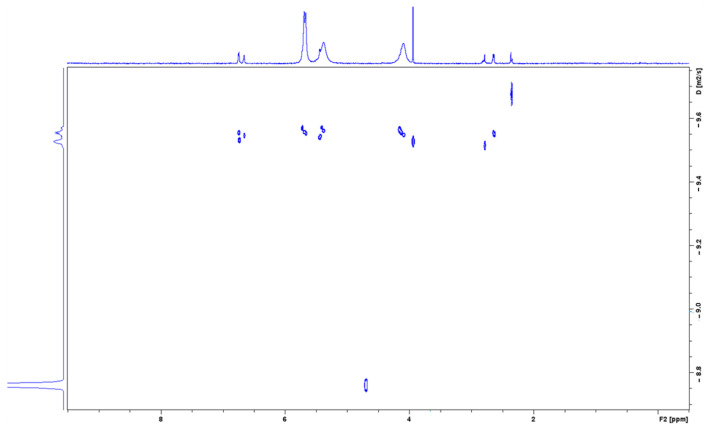
A part of the DOSY spectum of the NAB-CB7 1:1 complex in D_2_O at 25 °C.

To confirm the formation of the NAB-CB7 complex, the DOSY ^1^H NMR spectra of the NAB-CB7 solution were also recorded. As shown in Figure 8, different diffusion coefficients were detected in the DOSY NMR spectra, indicating the presence of different components in the solution, including the NAB-CB7 complex (D = 2.66 × 10^−10^ m^2^/s), non-associated CB7 (D = 2.72 × 10^−10^ m^2^/s), and non-associated NAB (D = 3.24 × 10^−10^ m^2^/s), respectively. The small difference in the diffusion coefficients between CB7 and the complex is a consequence of the small size difference between the two compounds.

**Figure 9:**
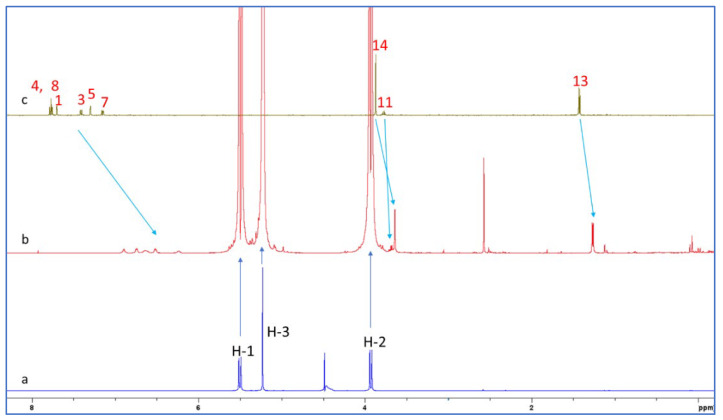
^1^H NMR spectra of (**a**) CB7; (**b**) NAP-CB7 1:1 complex; and (**c**) NAP in pure D_2_O at 25 °C.

The ^1^H NMR spectra of CB7, the NAP-CB7 1:1 complex, and NAP in D_2_O are shown in Figure 9. An analysis of these spectra showed a significant change in the chemical shift of NAP after complexation, similar to the changes observed for the NAB-CB7 system. As can be seen from Figure 9 and Table 2, all the NAP protons were shifted to lower ppm values after complexation. The largest chemical shift after complexation with CB7 is observed for the naphthenic protons (Table 2). This indicates that these protons are located deep inside the CB7 cavity, and that the observed upfield shift is related to the shielding effect of the hydrophobic cavity of CB7 [31]. Three resonance protons of the singlet OCH3, quartet CH, and doublet CH3 units were shifted to lower ppm values, which is in contrast to the previously reported data [24]. These results indicate that the NAP molecule was encapsulated in the hydrophobic cavity of CB7 in the same way as the NAB molecule. In addition, 2D ROESY experiments of NAP-CB7 1:1 were performed. The analysis of the ROESY spectra revealed only the intramolecular crosspeaks of CB7 (Figure S14).

In an attempt to determine the binding constants of NAP and NAB with CB7, we performed NMR titrations according to the published method [24]. The concentration of NAP and NAB was kept constant (1.5 mM), and the concentration of CB7 was gradually increased (0.5 mM; 1.0 mM; 1.5 mM, 2.0 mM; 2.5 mM). Although the formation of the complexes was observed in the ^1^H NMR spectra, it was not possible to determine the exact stability constant. Details can be found in the SI (Figures S15 and S16, Tables S5 and S6). Our results are not in agreement with the study by Meetani et al., [24] in which they reported the formation of a stable NAP-CB7 1:1 complex with a binding constant of (1.9 ± 0.3) × 10^6^ M^−1^ at approximately pH 6, as determined by ^1^H NMR titration. Considering that NAP is predominantly present as an anion at pH 6, this value of the binding constant is unlikely due to the strong electrostatic repulsion between negatively charged NAP and partially negatively charged CB7 portals [34].

### 2.3. Isothermal Titration Calorimetry

Isothermal titration calorimetry is an important tool for the thermodynamic investigation of intermolecular interactions. It is based on the measurement of the heat generated or absorbed during the interaction between two molecules. ITC provides valuable information about the binding affinity, the binding stoichiometry, and the driving forces for the reaction under investigation. In addition, ITC analysis can be performed at very low concentrations, making it particularly suitable for poorly soluble compounds, such as nabumetone.

**Figure 10:**
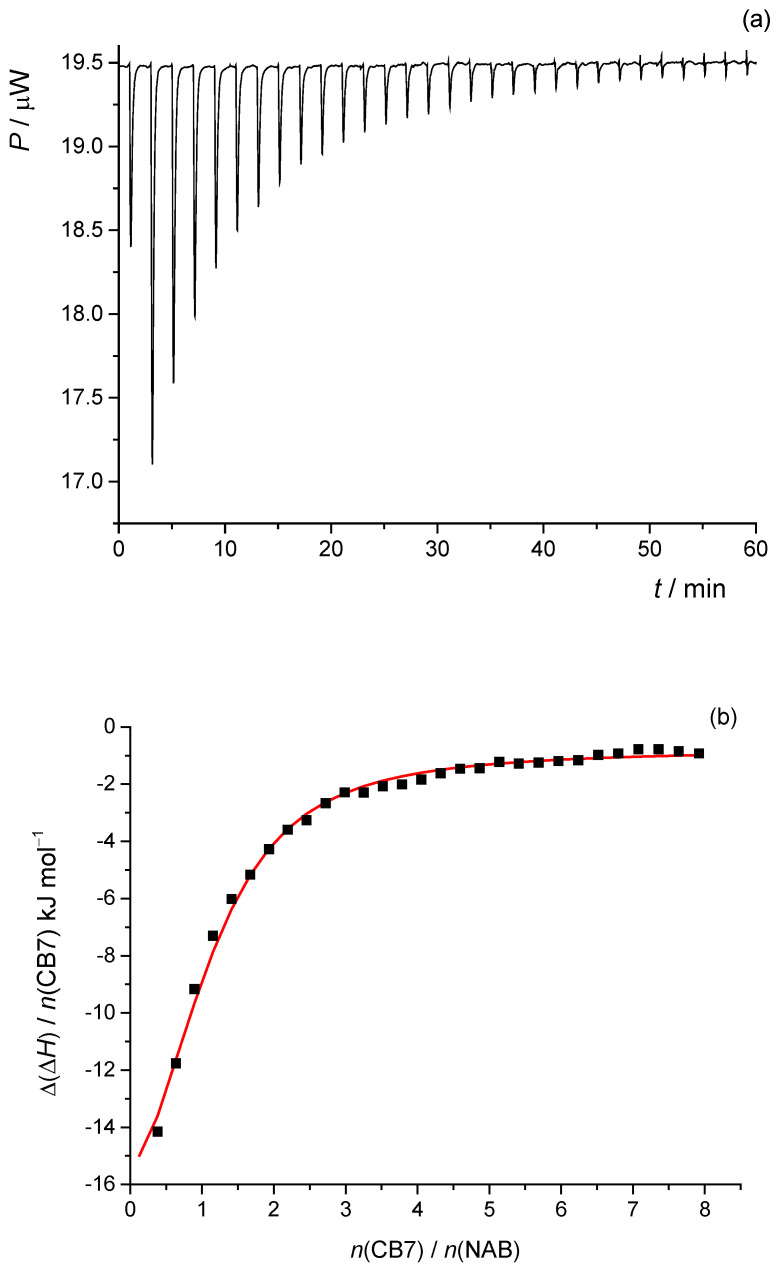
(**a**) Microcalorimetric titration of NAB (*c*_0_ = 48.9 × 10^−6^ mol L^−1^) with CB7 (*c*_0_ = 3.81 × 10^−3^ mol L^−1^) in water at 298 K. (**b**) Dependence of normalized successive enthalpy changes on CB7/NAB molar ratio. ■ means experimental; ― means calculated.

To investigate the complexation reaction between NAB and CB7, isothermal microcalorimetric titrations were performed in pure water at 25 °C (Figure 10a). The normalized successive enthalpy changes with respect to the molar ratio CB7/NAB (Figure 10b) were consistent with the formation of a 1:1 complex. By processing the calorimetric data accordingly, the standard thermodynamic parameters of the complexation reaction (reaction enthalpy, entropy, and Gibbs free energy) and the corresponding equilibrium constant were determined (Table 3).

Like most cyclodextrin cases, cucurbituril binding is mainly driven by favorable enthalpic gains, accompanied by either positive or negative entropic changes. The values of the thermodynamic parameters show that the binding of NAB is both enthalpically and entropically favorable, but the absolute value of Δ_r_*H*° is higher than the absolute value of *T*Δ_r_*S*°. The complexation process of NAB with CB7 is exothermic, which is expected and common for cucurbiturils [35,36]. The water molecules within the CB7 cavity are structurally and energetically disadvantaged, e.g., they have a lower number of hydrogen bonds than in bulk water [35]. Since these molecules are released from the cavity during the complexation reaction, the binding is enthalpically favorable. The value of the reaction entropy is positive. Entropy is a complex quantity, whose value is the result of several contributions, including the desolvation entropy of the guest molecule upon inclusion in the host and the release of water molecules from the cavity. Since the release of water molecules is predicted to be entropically unfavorable, the desolvation entropy is the dominant force associated with the binding process [37,38]. The desolvation process is considered entropically favorable. If the entropic effect of the desolvation of the drug outweighs that of the release of water molecules from the cucurbituril cavity, the reaction entropy will be positive, as is the case for the herein-studied inclusion reaction.

As can be seen from the data presented in Table 3, the calculated stability constant (log *K* = 4.66) indicates that CB7 is an effective binder for NAB. This value is more than an order of magnitude higher than the values of the stability constants for the equivalent complexes of NAB with β-CDs [19]. This is due to the more rigid structure of cucurbiturils compared to the more flexible cyclodextrins, which allows them to form a more precise fit with their guests. This precise fit enhances the binding strength and leads to higher stability constants. In addition, cucurbiturils are very shape-selective. The size and shape of the guest must match the cavity, which leads to a more specific interaction and, therefore, a higher stability constant [39].

Although the ^1^H NMR, HR MS as well as the theoretical calculations, indicated the formation of an inclusion complex between CB7 and naproxen, no significant complex formation between CB7 and NAP was observed in the ITC experiments, neither at pH 6.8 nor at pH 2 (Figures S17 and S18). This behavior can be explained by considering the physicochemical properties of the host and the guest at different pH values. At a pH of 6.8, NAP is predominantly present as a carboxylate anion, resulting in significant electrostatic repulsion with the negatively charged, carbonyl-lined portals of CB7 [34]. This repulsion likely prevents the effective penetration of the guest into the host cavity. In addition, the strong solvation shell around the carboxylate group increases the desolvation energy, which is not sufficiently compensated by the interactions between CB7 and the guest [34]. At an acidic pH, NAP becomes protonated and neutral, possibly abolishing the charge-based repulsion at the CB7 portals. However, no significant complex formation is observed, even under these conditions. This can be attributed to several factors. The condensed aromatic system and the short side chain of NAP result in a relatively rigid and flat molecule that does not sterically fit into the cavity of CB7. Furthermore, a neutral NAP does not have a strong cationic center and can only form weak hydrophobic interactions that are insufficient to drive complex formation under competitive acidic conditions, where high concentrations of H_3_O^+^ compete effectively with CB7 binding (log *K*(H_3_O^+^) = 2.22 [30]) and reduce the affinity for NAP. In addition, NAP can self-associate under acidic conditions, which could further reduce the availability of monomeric guest molecules for incorporation.

### 2.4. Computational Analysis

Computational chemistry methods are a valuable tool for identifying and characterizing dominant binding interactions during the inclusion of non-polar molecules into the hydrophobic cavity of a host molecule. In aqueous solution, the contributions to inclusion come from electrostatic interactions, hydrogen bonding, dispersion interactions, and hydrophobic effects. Computational methods have been developed, with which these very weak interactions can be investigated [40]. Among others, the independent gradient model, based on the Hirshfeld partition of a molecular density (IGMH) analysis, has proven to be a useful visual method for the detection of intermolecular, noncovalent interactions [40,41]. In an IGMH analysis, the density gradient difference (*δg*^inter^) isosurfaces are colored with *sign*(*λ*_2_)*ρ* values to visually illustrate weak attractive interactions, such as hydrogen bonds, van der Waals interactions, and weak repulsive steric interactions [41].

**Figure 11:**
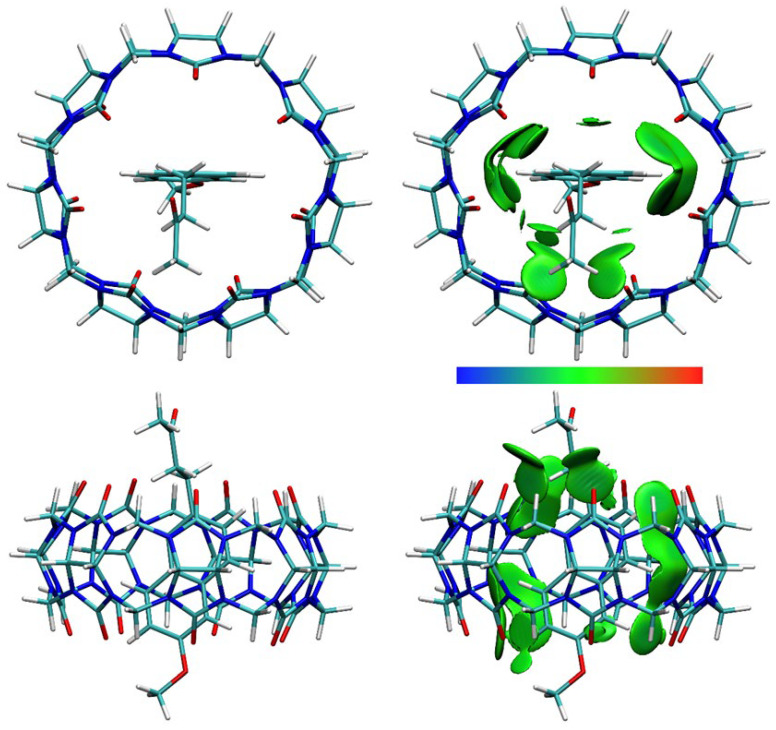
The structure (**left**) and the IGMH analysis (**right**) of the NAB-CB7 1:1 host–guest complex. In the IGMH analysis, the isosurfaces of the *δg*^inter^ = 0.002 a.u. are colored with *sign*(*λ*_2_)*ρ* in the range of −0.05 (blue), 0.00 (green), and 0.05 (red), revealing weak attractive interactions, van der Waals interactions, and repulsive interactions, respectively.

**Figure 12:**
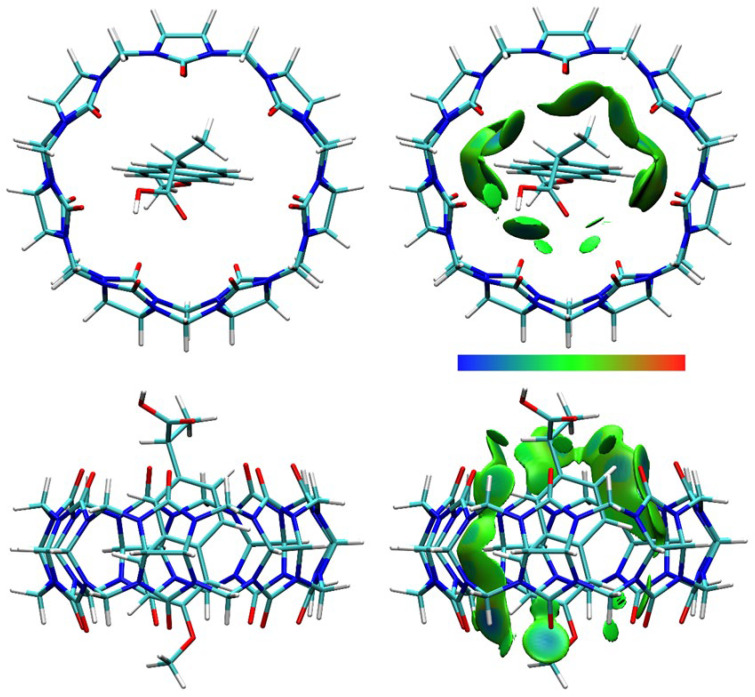
The structure (**left**) and the IGMH analysis (**right**) of the NAP(acid)–CB7 1:1 host–guest complex. In the IGMH analysis, the isosurfaces of the *δg*^inter^ = 0.002 a.u. are colored with *sign*(*λ*_2_)*ρ* in the range of −0.05 (blue), 0.00 (green), and 0.05 (red), revealing weak attractive interactions, van der Waals interactions, and repulsive interactions, respectively.

**Figure 13:**
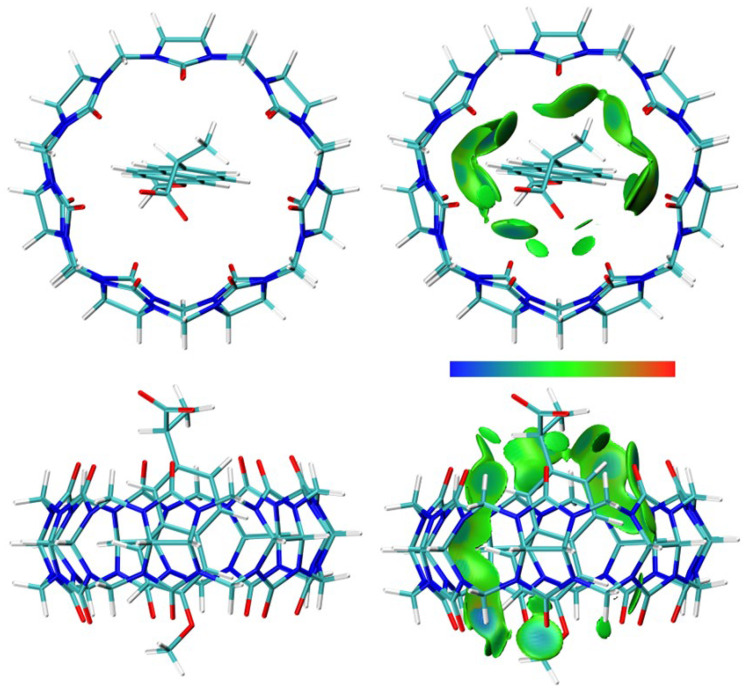
The structure (**left**) and the IGMH analysis (**right**) of the NAP(anion)–CB7 1:1 host–guest complex. In the IGMH analysis, the isosurfaces of the *δg*^inter^ = 0.002 a.u. are colored with *sign*(*λ*_2_)*ρ* in the range of −0.05 (blue), 0.00 (green), and 0.05 (red), revealing weak attractive interactions, van der Waals interactions, and repulsive interactions, respectively.

DFT calculations were performed, and optimized structures were obtained for NAB-CB7, NAP(acid)–CB7, and NAP(anion)–CB7 1:1 host–guest systems, where the aromatic rings of the guests are encapsulated within the CB7 cavity (Figure 11, Figure 12 and Figure 13, Figures S19–S24). The distances between the aromatic H atoms of the guests and the H atoms of CB7 are 3.20 Å–3.83 Å and are comparable to the distances of 3.31 Å–3.84 Å obtained for a similar aromatic host–guest system [42]. The calculated dipole moment of NAB, NAP (acid), and NAP (anion) are 4.35, 4.47, and 22.20 Debyes, respectively. The guests are only partially embedded in the cavity, and the methyl, carboxyl, and methoxy groups protrude beyond the CB7 host (Figure 11, Figure 12 and Figure 13). The volume of the immersed part of the guests can be estimated to be 136 Å^3^ [43], and, taking into account the calculated volume of the CB7 inner cavity of 242 Å^3^ [44], the packing coefficient (PC) of the host–guest systems is 56%. This obtained PC value is close to the Rebek’s 55% packing rule, suggesting a very high binding affinity [44,45].

The calculated binding Gibbs energies for NAB-CB7, NAP (acid)–CB7, and NAP (anion)–CB7 1:1 host–guest systems, after correction to the 1 M reference states [46] of −39.7, −16.8, and −0.3 kJ mol^−1^, respectively, are in reasonable agreement with the experimentally obtained values. Correspondingly similar values were obtained by quantum chemical calculations for other host–guest inclusion systems in water, where it was also shown that an error of this order of magnitude exists due to the hydrophobic effect, which is not accounted for in the applied calculation methods [46,47]. In part, the hydrophobic effect can be estimated by assuming that a certain number of water molecules are initially present in the CB7 cavity and are displaced from the cavity in the inclusion reaction. The number of water molecules inside the CB7 cavity is about eight, as estimated by several methods [44,48]. The calculated Gibbs energy for displacing eight water molecules structured within the CB7 cavity is +2.1 kJ mol^−1^ and +38.9 kJ mol^−1^ depending on the model, i.e., whether the released water retains the ordered structure or not [48]. Approximately half of the water molecules tend to form clusters of various sizes in the bulk water, so the Gibbs energy for the process of displacing water molecules from the CB7 cavity is expected to be within this range. Also, a further correction to the calculated binding Gibbs energy for the host–guest systems could be of that magnitude.

To obtain structural information about the host–guest systems, an IGMH analysis was performed on the optimized structures. The IGMH analysis of the complexes shows that weak van der Waals interactions play a major role in the interaction of the hydrophobic cavity of CB7 with the aromatic rings and chains of the guests (Figure 11, Figure 12 and Figure 13, Figures S19–S24), which is also consistent with the ^1^H NMR data. The (3, −1) bonds’ critical points (BCPs), obtained by an atoms-in-molecules (AIM) analysis, corresponding to these interactions are listed in Tables S8–S10 and shown in Figures S19–S24. The found BCPs can be attributed to the O∙∙∙H, N∙∙∙H and O∙∙∙Ar contacts. The values for density (*ρ*) and the density gradient difference (*δg*) obtained for these BCPs are very small, indicating very weak intermolecular interactions.

## 3. Materials and Methods

### 3.1. Materials

All the chemicals were used as received without further purification. Nabumetone and naproxen were purchased from Cayman Chemical (Ann Arbor, MI, USA). Cucurbit[7]uril hydrate was purchased from Sigma Aldrich (St. Louis, MO, USA) and used as received. The concentration was corrected by a water content of 20%, as specified by the supplier. The methanol (MeOH) and formic acid (FA) were LC-MS grade and purchased from Carlo Erba (Milan, Italy). The ammonium formate was LC-MS grade and purchased from Sigma Aldrich. Ultrapure water was obtained using the Mili-Q Advantage A10 purification system (Merck, Darmstadt, Germany). Deuterated D_2_O was purchased from Eurisotop (Cambridge Isotope Laboratories, Inc., Saint-Aubin Cedex, France).

### 3.2. Mass Spectrometry

High-resolution mass spectra were acquired on an Agilent 6550 Series Accurate-Mass Quadrupole Time-of-Flight (Q-TOF) (Agilent, Santa Clara, CA, USA). Initially, solutions were introduced directly via an Agilent 1290 Infinity II UHPLC ((Agilent, Santa Clara, CA, USA)) using flow injection analysis. The mobile phase consisted of 0.1% formic acid in MeOH (A) and 0.1% formic acid in H_2_O (B) in an isocratic mode at a ratio of 80:20 (*v/v*). The flow rate was set to 0.4 mL min^−1^ with an injection volume of 5 μL. Subsequently, to enhance the abundance of complex molecular ions, the samples were directly infused into the electrospray ionization source at a flow rate of 10 μL min^−1^ using a KD Scientific syringe pump (KD Scientific, Holliston, MA, USA). Between each analysis, the syringe and the ESI capillary were cleaned with a solution of water, methanol, acetonitrile, and isopropanol (1:1:1:1) containing 2% formic acid. The ESI–MS spectra were obtained in positive (ESI+) and negative (ESI−) ion mode, ranging from *m/z* 100 to *m/z* 1500. The fine-tuning of the ESI ionization source parameters was performed to achieve higher abundances of complex molecular ions in the MS spectra. For the ESI+ mode, the capillary voltage was 3000 V, fragmentor voltage 100 V, drying gas flow 15 dm^3^ min^−1^, and temperature 225 °C. The sheath gas flow was 10 dm^3^ min^−1^ and the temperature was 200 °C. The nozzle voltage was 1500 V. For the ESI− analyses, the drying gas and sheath gas temperatures were set to 200 and 325 °C, respectively, and the flow was set to 20 and 11 dm^3^ min^−1^. Nitrogen was used as a drying and sheath gas. For the representative MS/MS spectra, the collision energy (CE) varied from 5 to 15 or 20 eV. The doubly charged complex molecular ions were subjected to CID analyses (four determinations) at incremental CE values ranging from 0 to 16 eV, where 5 scans were accumulated for each analysis. The relative intensities (RI) of the precursor ions at each applied collision energy were calculated from the absolute intensities of the complex *I*_HG_ and the host *I*_H_, based on Equation (1), which was proposed by Gabelica et al. [49], with modifications. The CE_50_ values were derived from the plots of the calculated RI vs. the applied CE by using a fit with a sigmoidal Boltzmann function (OriginPro 2016).

(1) $$RI\;\left(\%\right)=\frac{I_{HG}}{I_{HG}+\frac{I_{H}}{2}}\times 100\%$$

Stock solutions of NAB and NAP were prepared in methanol, while CB7 was prepared in H_2_O. Complex solutions with a molar ratio of *n*(NAB/NAP):*n*(CB7) = 1:1 were prepared in either methanol–water (with 0.1% formic acid) or methanol–5 mM ammonium formate (with 0.1% formic acid) at a volume ratio of 10:90 (*v*/*v*). The final concentration of NAB or NAP in measured solutions was 2.15 × 10^−5^ mol L^−1^.

### 3.3. NMR Spectroscopy

NMR spectra were aquired on a Bruker Avance AV600 spectrometer (Bruker, Rheinstetten, Germany) equipped with a room-temperature 5 mm BBO probe with a z-gradient accessory using standard Bruker pulse sequences in D_2_O at 25 °C. An NMR analysis was based on ^1^H, ROESY, and DOSY NMR techniques, and the WaterGate scheme was used to suppress the HDO signal. The data were processed using the Bruker TopSpin 4.2.0. software package (Bruker, Rheinstetten, Germany). The 2D ROESY of the NAB-CB7 1:1 and the NAP-CB7 1:1 spectra were obtained with a mixing time of 500 ms. The DOSY NMR spectrum of the NAB-CB7 1:1 was recorded using dstebpgp3s, a pseudo-2D sequence with a double-simulated echo for convection compensation and longitudinal encode–decode (LED) with bipolar gradient pulses for diffusion and three spoil gradients. For the NMR analysis of NAB-CB7 1:1, 2.05 mg of NAB and 10.5 mg of CB7 were mixed and dissolved in 600 µL of D_2_O to achieve a concentration of 15 mmol L^−1^, while for the NMR analysis of NAP-CB7 1:1, 1.38 mg of NAP and 6.98 mg of CB7 were mixed and dissolved in 600 µL of D_2_O to achieve a concentration of 10 mmol L^−1^. The samples of NAB and CB7 were also prepared in D_2_O at a concentration of 15 mmol L^−1^ and NAP at a concentration of 10 mmol L^−1^. The exact final concentrations were not determined as all the samples (NAB, CB7, NAB-CB7, and NAP-CB7) precipitated in the D_2_O solution.

### 3.4. Isothermal Titration Microcalorimetry

The affinity between NAB and CB7 was measured by isothermal titration calorimetry using a PEAQ-ITC MicroCal isothermal titration calorimeter (Malvern Panalytical Ltd., Malvern, UK) at 25 °C. All the solutions were prepared in purified water (Milli-Q, Millipore) and briefly degassed before use. The CB7 solution (3.18 × 10^−3^ mol L^−1^) was titrated to NAB (*V*_0_ = 0.20 mL; *c*_0_ = 4.89 × 10^−5^ mol L^−1^) in 30 injections (the first injection was 0.4 μL, then 0.8 μL each) with an injection interval of 150 s while stirring at 750 rpm. The enthalpy changes measured in the titration experiments were corrected for the heats corresponding to the dilution of the CB7 solutions obtained by the blank experiments. The dependence of the successive enthalpy changes on the titrant volume was analyzed using a single binding site model, which is available in MicroCal PEAQ-ITC Analysis software (version 1.41). The stoichiometry was set to 1.00 in the fitting procedure because the binding stoichiometry was determined in separate experiments (HR MS). The first data point was always removed. The titrations were repeated 3 times (Table S7).

Since the solubility of NAP is low and strongly dependent on the pH, the ITC experiments were performed at pH 2 and at pH 6.8, where the drug should be neutral and negatively charged, respectively. The solutions were prepared in 0.01 mol dm^−3^ aqueous HCl or in phosphate buffer. At pH 2, the CB7 solution (3.4 × 10^−3^ mol L^−1^) was titrated in 13 injections of 3 μL to NAP (*V*_0_ = 0.20 mL; *c*_0_ = 6.3 × 10^−5^ mol L^−1^) with an injection spacing of 150 s while stirring at 750 rpm. At pH 6.8, the CB7 solution (11.4 × 10^−3^ mol L^−1^) was titrated in 19 injections of 2 μL to NAP (*V*_0_ = 0.20 mL; *c*_0_ = 1.83 × 10^−3^ mol L^−1^) with an injection spacing of 150 s while stirring at 750 rpm. The resulting thermogram showed no significant heat change above the background levels across the injections, and the binding isotherm was flat, indicating no detectable enthalpic signal associated with a binding event (Figures S17 and S18).

### 3.5. Computational Methods

The calculations were performed using GAUSSIAN 16 software [50] at the B3LYP/6-311+G (2d,2p) level [51,52] with the D3 version of Grimme’s empirical dispersion correction [53]. Non-specific solvent effects were evaluated using the polarizable continuum model (PCM) of the self-consistent reaction field (SCRF) method [54] with water as a solvent. The geometry optimization of the host–guest system conformers was performed, and the obtained stationary points were confirmed as minima by a vibrational analysis at the same theoretical level. An independent gradient model based on the Hirshfeld partition of molecular density (IGMH) analysis [41] and the atoms-in-molecules (AIM) topological analysis [55] were performed on the obtained wave functions of the host–guest system with the MULTIWFN 3.8 program [56,57] and plotted with the VMD 1.9.4 program [58].

## 4. Conclusions

In this study, we investigated the formation of supramolecular host–guest complexes between cucurbit[7]uril and two structurally related NSAIDs, nabumetone and naproxen. Our results show that NAB forms a stable 1:1 inclusion complex with CB7 in an aqueous solution, with a binding constant (log K = 4.66) that exceeds the affinity observed for β-cyclodextrins, highlighting the potential of CB7 as an efficient host for NAB. Thermodynamic analyses showed that the complex formation is both enthalpically and entropically favorable. In contrast, CB7 showed a significantly lower affinity for NAP in both its neutral and anionic forms, emphasizing the crucial role of the functional groups and the acid–base properties of guest molecules in complex formation with CB7. The nature of the NAB-CB7 and NAP-CB7 interactions and the structure of the complexes were proposed based on the results of the NMR experiments and the theoretical calculations. The results of this study are consistent with studies on the complexation of NAB and NAP with β-cyclodextrins, which show that β-cyclodextrins are a better binder for NAB than NAP [59,60].

## Figures and Tables

**Table 1:** Comparison of ^1^H NMR chemical shifts of NAB and NAB-CB7 1:1 complex in D_2_O at 25 °C.

Proton	NAB δ/ppm	NAB-CB7 1:1 δ/ppm
1	7.62	6.56–6.85
3	7.36	6.56–6.85
4	7.76	6.56–6.85
5	7.29	6.56–6.85
7	7.15	6.56–6.85
8	7.73	6.56–6.85
11	2.97	2.79
12	2.91	2.63
14	2.13	2.35
15	3.87	3.93

**Table 2:** Comparison of ^1^H NMR chemical shifts of NAP and NAP-CB7 1:1 complex in D_2_O at 25 °C.

Proton	NAP δ/ppm	NAP-CB7 1:1 δ/ppm
1	7.70	6.20–6.96
3	7.41	6.20–6.96
4	7.77	6.20–6.96
5	7.30	6.20–6.96
7	7.16	6.20–6.96
8	7.77	6.20–6.96
11	3.77	3.69
13	1.43	1.23
14	3.88	3.65

**Table 3:** Equilibrium constant (log *K*) and thermodynamic parameters for complexation of NAB with CB7 at 25 °C in water.

Complex	$\log\left(\frac{K}{{\mathrm{dm}}^{3}{\;\mathrm{mol}}^{-1}}\right)\pm \mathrm{SE}$	$\frac{\left(\Delta_{r}G{}^{\circ}\;\pm \;\mathrm{SE}\right)}{{\mathrm{kJ}\;\mathrm{mol}}^{-1}}$	$\frac{\left(\Delta_{r}H{}^{\circ}\;\pm \;\mathrm{SE}\right)}{{\mathrm{kJ}\;\mathrm{mol}}^{-1}}$	$\frac{\left(T\Delta_{r}S{}^{\circ}\;\pm \;\mathrm{SE}\right)}{{\mathrm{kJ}\;\mathrm{mol}}^{-1}}$
NAB-CB7	4.66 ± 0.01	−26.7 ± 0.1	−20.3 ± 0.9	6.3 ± 0.9

SE—standard error of the mean (*N* = 3).

## Data Availability

The original contributions presented in this study are included in the article/Supplementary Materials. Further inquiries can be directed to the corresponding author.

## Supplementary Materials

The following supporting information can be downloaded at: https://www.mdpi.com/article/10.3390/molecules30122558/s1, Figure S1. ESI+ HRMS spectrum of cucurbituril7 solution (*c* = 2.15 × 10^−5^ M). Figure S2. MS/MS spectrum of [CB7+2NH_4_]^2+^ ion (*m*/*z* 599.2037) at 10 V. Table S1. Relative intensities and *m*/*z* values of signals in MS/MS spectra of [CB7+2NH_4_]^2+^ ion (*m*/*z* 599.2037) at 5, 10 and 20 eV. Figure S3. ESI+ HRMS spectrum of cucurbituril7 solution (*c* = 2.15 × 10^−5^ M). Table S2. Relative intensities and *m*/*z* values of signals in HRMS spectra of nabumetone and cucurbituril7 solution in molar ratio 1:1. *c*(NAB) = 2.15 × 10^−5^ M. Table S3. Relative intensities and *m*/*z* values of signals in MS/MS spectra of [CB7/NAB+2NH_4_]^2+^ ion (*m*/*z* 713.2606) at 5, 10, and 15 V. Figure S4. MS/MS spectrum of [CB7/NAB+2NH_4_]^2+^ ion (*m*/*z* 713.2606) at 10 eV. Figure S5. MS/MS spectrum of [CB7/NAB+2NH_4_]^2+^ ion (*m*/*z* 713.2606) at 15 eV. Figure S6. MS/MS spectrum of [CB7/NAB+NH_4_+Na]^2+^ ion (*m*/*z* 715.7384) at 5 eV. Figure S7. MS/MS spectrum of [CB7/NAB+NH_4_+Na]^2+^ ion (*m*/*z* 715.7384) at 10 eV. Figure S8. MS/MS spectrum of [CB7/NAB+NH_4_+Na]^2+^ ion (*m*/*z* 715.7384) at 20 eV. Table S4. Relative intensities and *m*/*z* values of signals in HRMS spectra of nabumetone and cucurbituril7 solution in molar ratio 1:1. *c*(NAP) = 2.15 × 10^−5^ M. Figure S9. MS/MS spectrum of [CB7/NAP+2NH_4_]^2+^ ion (*m*/*z* 714.2500) at 5 eV. Figure S10. MS/MS spectrum of [CB7/NAP+2NH_4_]^2+^ ion (*m*/*z* 714.2500) at 10 eV. Figure S11. MS/MS spectrum of [CB7/NAP+2NH_4_]^2+^ ion (*m*/*z* 714.2500) at 15 eV. Figure S12. ESI– HRMS spectrum of naproxen and cucurbituril7 solution in molar ratio 1:1. c(NAP) = 2.15 × 10^−5^ M. Figure S13. ROESY spectrum of NAB-CB7 1:1 complex in D_2_O at 25 °C. Figure S14. Part of the ROESY spectrum showing intramolecular interactions of CB7 in D_2_O at 25 °C. Figure S15. ^1^H NMR spectra of (a) NAB (1.5 mM) (b) NAB (1.5 mM)–CB7 (0.5 mM) complex (c) NAB (1.5 mM)–CB7 (1.0 mM) complex (d) NAB (1.5 mM)–CB7 (1.5 mM) complex (e) NAB (1.5 mM)–CB7 (2.0 mM) complex (f) NAB (1.5 mM)–CB7 (2.5 mM) complex and (g) CB7 (10 mM) in D_2_O at 25 °C. Table S5. Comparision of ^1^H NMR chemical shifts of protons 11 and 12 of NAB (1.5 mM), NAB (1.5 mM)–CB7 (0.5 mM) complex, NAB (1.5 mM)–CB7 (1.0 mM) complex, NAB (1.5 mM)–CB7 (1.5 mM) complex, NAB (1.5 mM)–CB7 (2.0 mM) complex and NAB (1.5 mM)–CB7 (2.5 mM) complex in D_2_O at 25 °C. Figure S16. ^1^H NMR spectra of (a) NAP (1.5 mM) (b) NAP (1.5 mM)–CB7 (0.5 mM) complex (c) NAP (1.5 mM)–CB7 (1.0 mM) complex (d) NAP (1.5 mM)–CB7 (1.5 mM) complex (e) NAP (1.5 mM)–CB7 (2.0 mM) complex (f) NAP (1.5 mM)–CB7 (2.5 mM) complex and (g) CB7 (10 mM) in D_2_O at 25 °C. Table S6. Comparision of ^1^H NMR chemical shifts of protons 11 and 12 of NAP (1.5 mM), NAP (1.5 mM)–CB7 (0.5 mM) complex, NAP (1.5 mM)–CB7 (1.0 mM) complex, NAP (1.5 mM)–CB7 (1.5 mM) complex, NAP (1.5 mM)–CB7 (2.0 mM) complex and NAP (1.5 mM)–CB7 (2.5 mM) complex in D_2_O at 25 °C. Figure S17. (a) Microcalorimetric titration of NAP (*c*_0_ = 18.3 × 10^−5^ mol L^−1^) with CB7 (*c*_0_ = 11.4 × 10^−3^ mol L^−1^) at pH 6.8 (phosphate buffer) at 298 K. (b) Dependence of successive enthalpy changes on CB7/NAP molar ratio. Figure S18. (a) Microcalorimetric titration of NAP (*c*_0_ = 6.3 × 10^−5^ mol L^−1^) with CB7 (*c*_0_ = 3.4 × 10^−3^ mol L^−1^) at pH 2 (HCl) at 298 K. (b) Dependence of successive enthalpy changes on CB7/NAP molar ratio. Table S7. Equilibrium constant (log *K*) and thermodynamic parameters for complexation of NAB with CB7 at 25 °C in water. Figure S19. The structure and IGMH analysis (left) of NAB-CB7 1:1 host-guest complex. The isosurfaces of *δg*^inter^ = 0.002 a.u. are colored with *sign*(*λ*_2_)*ρ* in range from −0.05 (blue), 0.00 (green) and 0.05 (red), revealing weak attractive, van der Waals and repulsive interactions, respectively. The (3,−1) bond critical points (BCP, right) obtained by atoms-in-molecules (AIM) analysis of NAB-CB7 1:1 host-guest complex. Figure S20. The fingerprint plot obtained by IGMH analysis of NAB-CB7 1:1 host-guest complex. Figure S21. The structure and IGMH analysis (left) of NAP(acid)-CB7 1:1 host-guest complex. The isosurfaces of δ*g*^inter^ = 0.002 a.u. are colored with *sign*(*λ*_2_)*ρ* in range from −0.05 (blue), 0.00 (green) and 0.05 (red), revealing weak attractive, van der Waals and repulsive interactions, respectively. The (3,−1) bond critical points (BCP, right) obtained by atoms-in-molecules (AIM) analysis of NAP(acid)-CB7 1:1 host-guest complex. Figure S22. The fingerprint plot obtained by IGMH analysis of NAP(acid)-CB7 1:1 host-guest complex. Figure S23. The structure and IGMH analysis (left) of NAP(anion)-CB7 1:1 host-guest complex. The isosurfaces of *δg*^inter^ = 0.002 a.u. are colored with *sign*(*λ*_2_)*ρ* in range from −0.05 (blue), 0.00 (green) and 0.05 (red), revealing weak attractive, van der Waals and repulsive interactions, respectively. The (3,−1) bond critical points (BCP, right) obtained by atoms-in-molecules (AIM) analysis of NAP(anion)-CB7 1:1 host-guest complex. Figure S24. The fingerprint plot obtained by IGMH analysis of NPXan-CB7 1:1 host-guest complex. Table S8. The values of density (*ρ*) and density gradient difference (*δg*) in *a.u.* for (3,−1) bond critical points (BCP) obtained by atoms-in-molecules (AIM) analysis of NAB-CB7 1:1 host-guest complex. The BCP points and the corresponding numbers are shown in Figure S19 (right). Table S9. The values of density (*ρ*) and density gradient difference (*δg*) in *a.u.* for (3,−1) bond critical points (BCP) obtained by atoms-in-molecules (AIM) analysis of NAP(acid)-CB7 1:1 host-guest complex. The BCP points and the corresponding numbers are shown in Figure S21 (right). Table S10. The values of density (*ρ*) and density gradient difference (*δg*) in *a.u.* for (3,−1) bond critical points (BCP) obtained by atoms-in-molecules (AIM) analysis of NAP(anion)-CB7 1:1 host-guest complex. The BCP points and the corresponding numbers are shown in Figure S23 (right).
