# Supplementary material for: Host–Guest Interactions of Cucurbit[7]uril with Nabumetone and Naproxen: Spectroscopic, Calorimetric, and DFT Studies in Aqueous Solution
Source: Molecules. 2025 Jun 12;30(12):2558. doi: 10.3390/molecules30122558 (PMC12195925; doi:10.3390/molecules30122558)
Supplement: Supplementary file 1 [file molecules-30-02558-s001.zip › molecules-3464962-supplementary.pdf]

Host–guest interaction of cucurbit[7]uril with nabumetone and naproxen: Spectroscopic, calorimetric, and DFT studies in aqueous solution

David Klarić,[1] Valentina Borko,[2] Jelena Parlov Vuković,[3] Viktor Pilepić,[2] Ana Budimir[3*] and Nives Galić[1]

Supporting Information

Contents

1. **Mass spectrometry**
2. **NMR spectroscopy**
3. **Isothermal titration calorimetry**
4. **Computational details**

1. Mass spectrometry

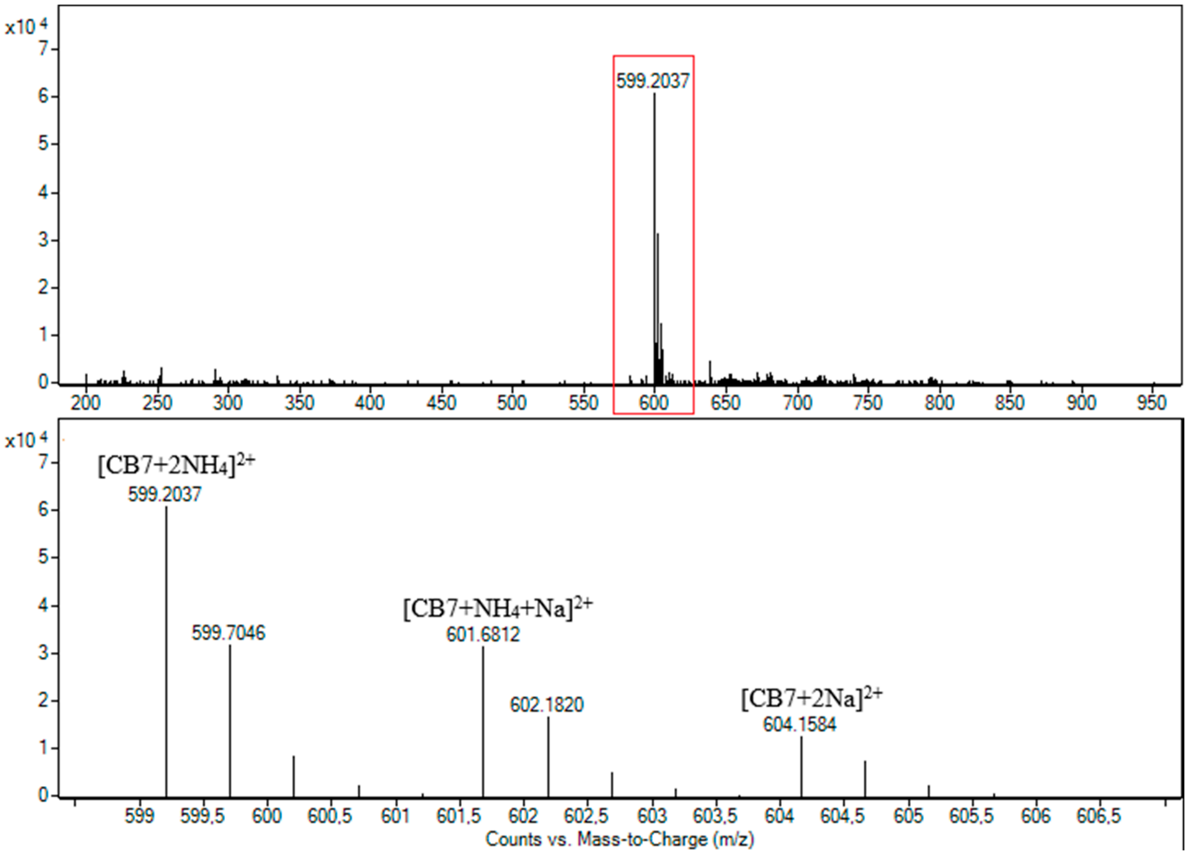

Figure S1. ESI+ HRMS spectrum of cucurbituril7 solution ($c = 2.15 \times 10^{-5}$ M).

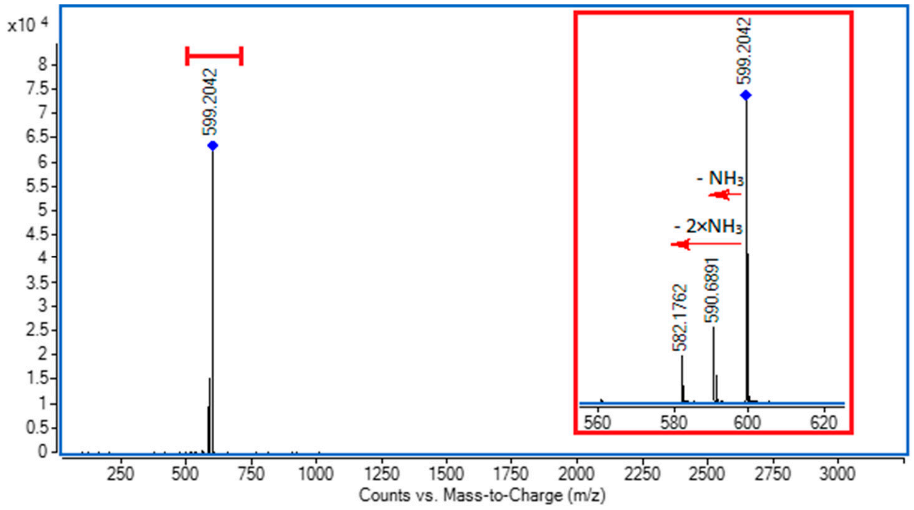

Figure S2. MS/MS spectrum of $[CB7+2NH_4]^{2+}$ ion (m/z 599.2037) at 10 V.

Table S1. Relative intensities and *m/z* values of signals in MS/MS spectra of $[CB7+2NH_4]^{2+}$ ion (*m/z* 599.2037) at 5, 10 and 20 eV.

5 eV		10 eV		20 eV		Assignation
m/z	rel.int / %	*m/z*	rel.int / %	*m/z*	rel.int / %	
599.2045	100.00	599.2042	100.00	599.2027	9.27	$[CB7+2NH_4]^{2+}$
590.6895	5.70	590.6891	23.71	590.6906	28.92	$[M–NH_3]^{2+}$
582.1755	4.33	582.1762	14.39	582.1776	100.00	$[M–2NH_3]^{2+}$

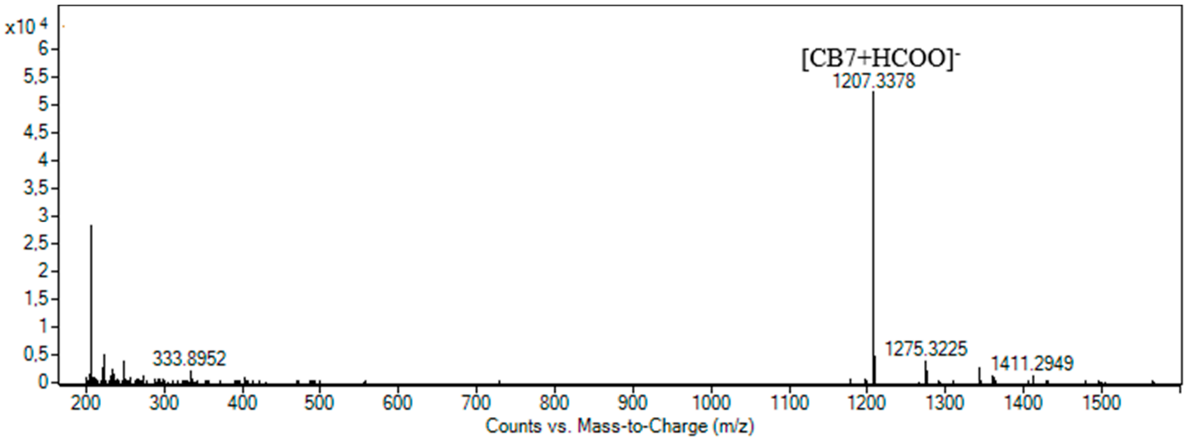

Figure S3. ESI+ HRMS spectrum of cucurbituril7 solution ($c = 2.15 \times 10^{-5}$ M).

Table S2. Relative intensities and *m/z* values of signals in HRMS spectra of nabumetone and cucurbituril7 solution in molar ratio 1:1. c(NAB) = 2.15×10^{-5} M

m/z exp.	*m/z* calc.	Mass diff. / ppm	Assignation
229.1232	229.1223	3.90	$[NAB+H]^{+}$
251.1036	251.1043	–2.59	$[NAB+Na]^{+}$
599.2042	599.2056	–2.32	$[CB7+2NH_4]^{2+}$
601.6822	601.6833	–1.81	$[CB7+NH_4+Na]^{2+}$
713.2606	713.2631	–3.51	$[CB7/NAB+2NH_4]^{2+}$
715.7384	715.7408	–3.36	$[CB7/NAB+NH_4+Na]^{2+}$
718.2150	718.2185	–4.87	$[CB7/NAB+2Na]^{2+}$

Table S3. Relative intensities and *m*/*z* values of signals in MS/MS spectra of $[CB7/NAB+2NH_4]^{2+}$ ion (*m*/*z* 713.2606) at 5, 10, and 15 V.

5 eV		10 eV		15 eV		Assignation
m/*z*	rel.int / %	*m*/*z*	rel.int / %	*m*/*z*	rel.int / %	
713.2596	100.00	713.2599	28.03	-	-	$[CB7/NAB+2NH_4]^{2+}$
704.7463	14.67	704.7464	22.31	704.7459	7.34	$[M–NH_3]^{2+}$
696.2363	1.14	696.2333	4.14	696.2332	6.06	$[M–2NH_3]^{2+}$
599.2025	29.35	599.2029	100.00	599.2032	100.00	$[M–NAB]^{2+}$
590.6904	1.13	590.6870	3.03	590.6895	6.16	$[M–NAB–NH_3]^{2+}$
582.1746	1.08	582.1753	2.04	582.1769	3.82	$[M–NAB–2NH_3]^{2+}$

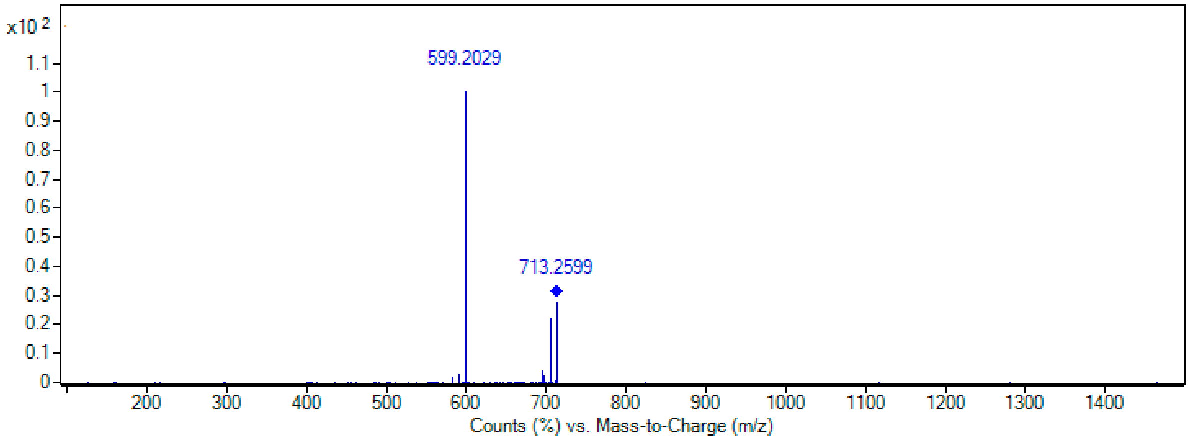

Figure S4. MS/MS spectrum of $[CB7/NAB+2NH_4]^{2+}$ ion (*m*/*z* 713.2606) at 10 eV.

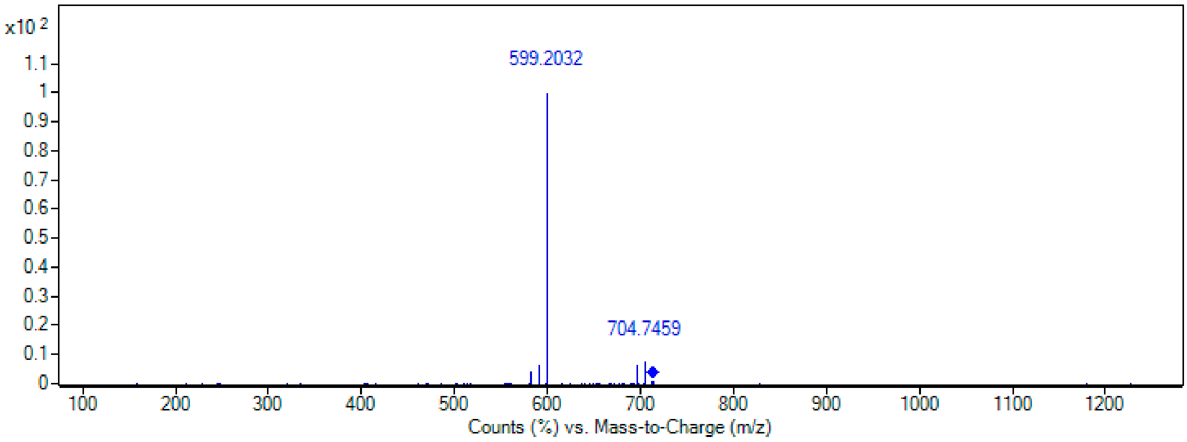

Figure S5. MS/MS spectrum of $[CB7/NAB+2NH_4]^{2+}$ ion (*m/z* 713.2606) at 15 eV.

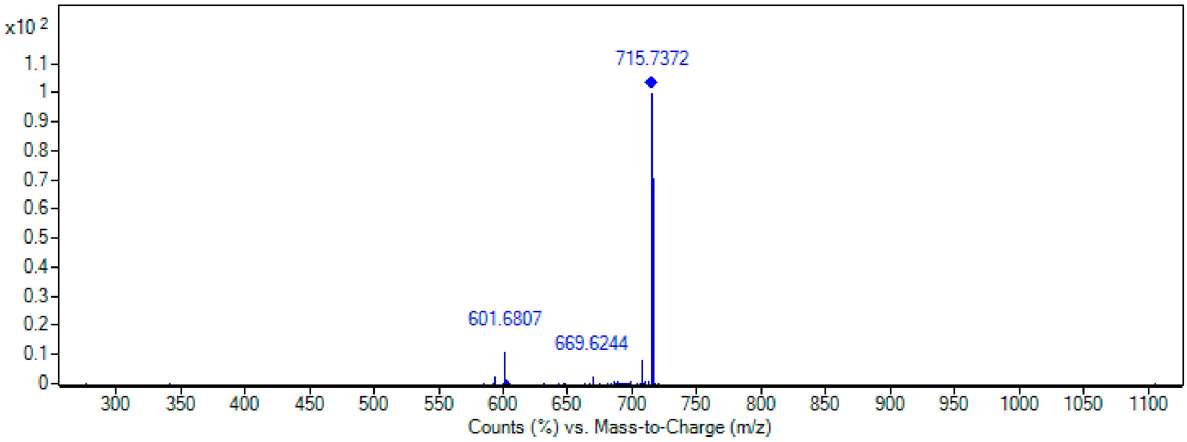

Figure S6. MS/MS spectrum of $[CB7/NAB+NH_4+Na]^{2+}$ ion (*m/z* 715.7384) at 5 eV.

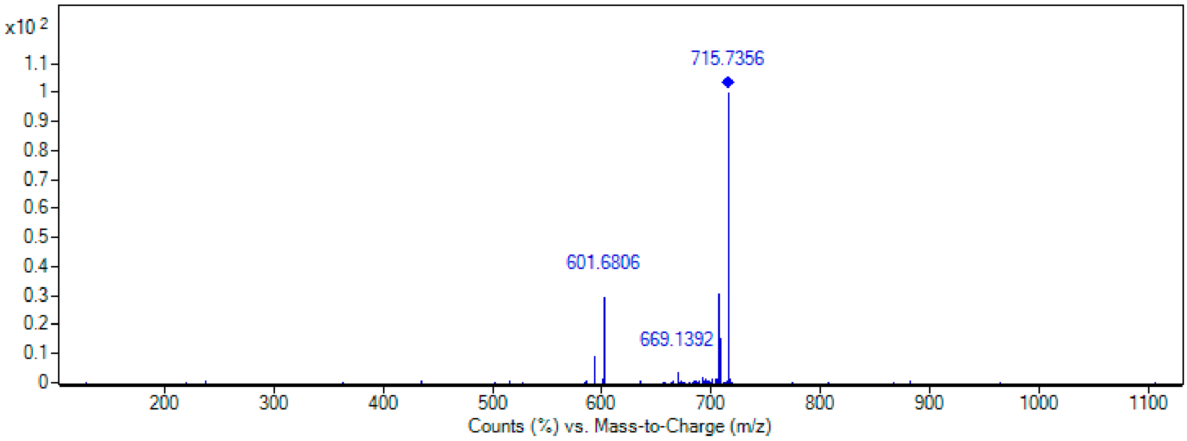

Figure S7. MS/MS spectrum of $[CB7/NAB+NH_4+Na]^{2+}$ ion (*m/z* 715.7384) at 10 eV.

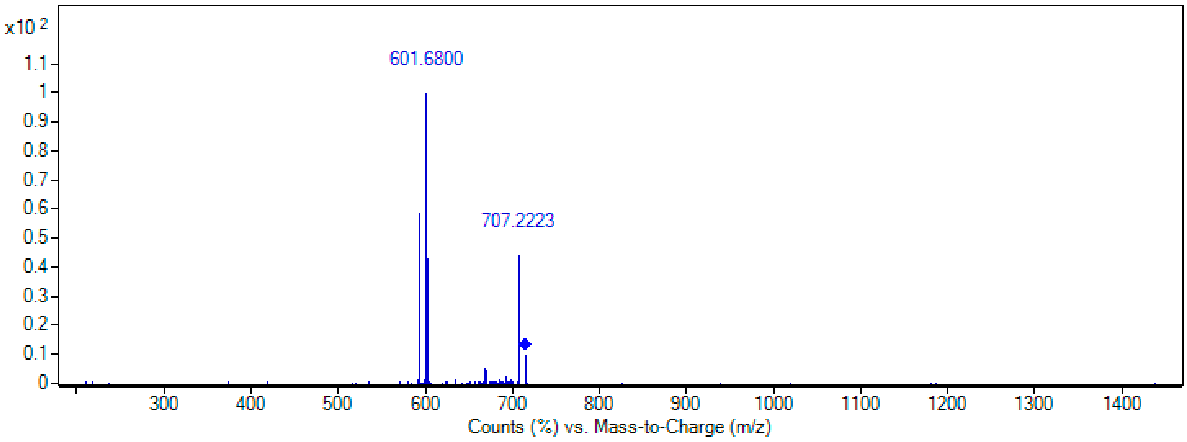

Figure S8. MS/MS spectrum of $[CB7/NAB+NH_4+Na]^{2+}$ ion (m/z 715.7384) at 20 eV.

Table S4. Relative intensities and m/z values of signals in HRMS spectra of nabumetone and cucurbituril7 solution in molar ratio 1:1. c(NAP) = 2.15×10^{-5} M

m/z exp.	m/z calc.	Mass diff. / ppm	Assignation
231.1016	231.1016	0.13	$[NAP+H]^{+}$
253.0830	253.0835	–2.04	$[NAP+Na]^{+}$
599.2045	599.2056	–1.82	$[CB7+2NH_4]^{2+}$
601.6817	601.6833	–2.64	$[CB7+NH_4+Na]^{2+}$
714.2500	714.2527	–3.83	$[CB7/NAP+2NH_4]^{2+}$
716.7274	716.7304	–4.23	$[CB7/NAP+NH_4+Na]^{2+}$
719.2062	719.2081	–2.69	$[CB7/NAP+2Na]^{2+}$

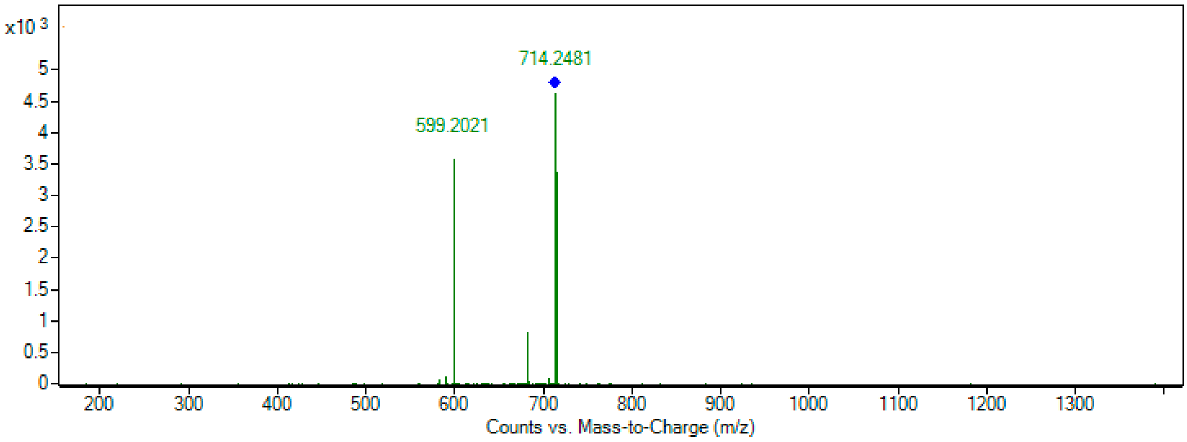

Figure S9. MS/MS spectrum of $[CB7/NAP+2NH_4]^{2+}$ ion (*m/z* 714.2500) at 5 eV.

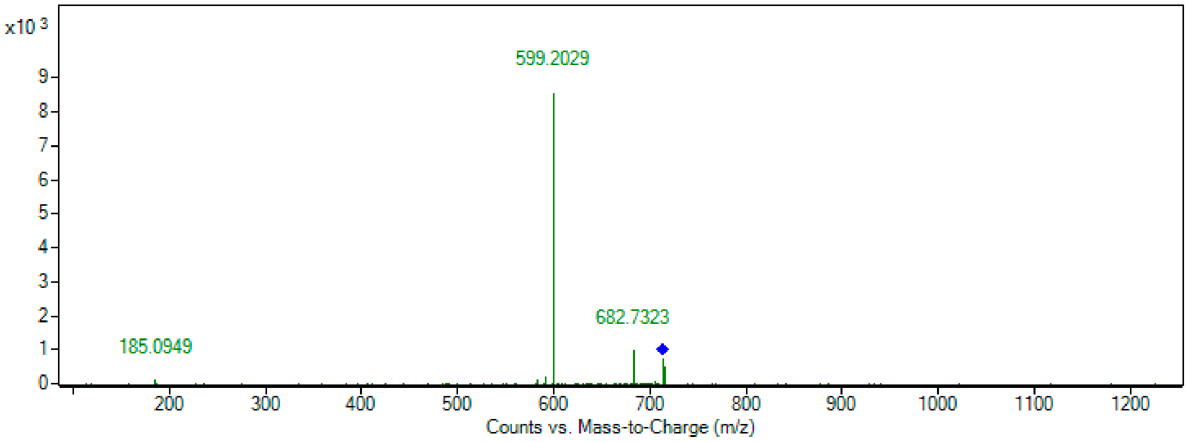

Figure S10. MS/MS spectrum of $[CB7/NAP+2NH_4]^{2+}$ ion (*m/z* 714.2500) at 10 eV.

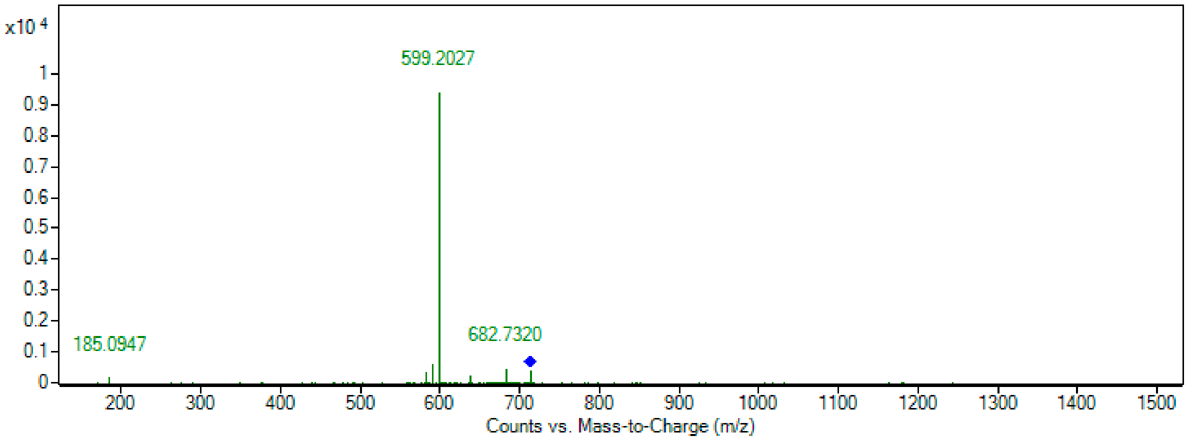

Figure S11. MS/MS spectrum of $[CB7/NAP+2NH_4]^{2+}$ ion (*m/z* 714.2500) at 15 eV.

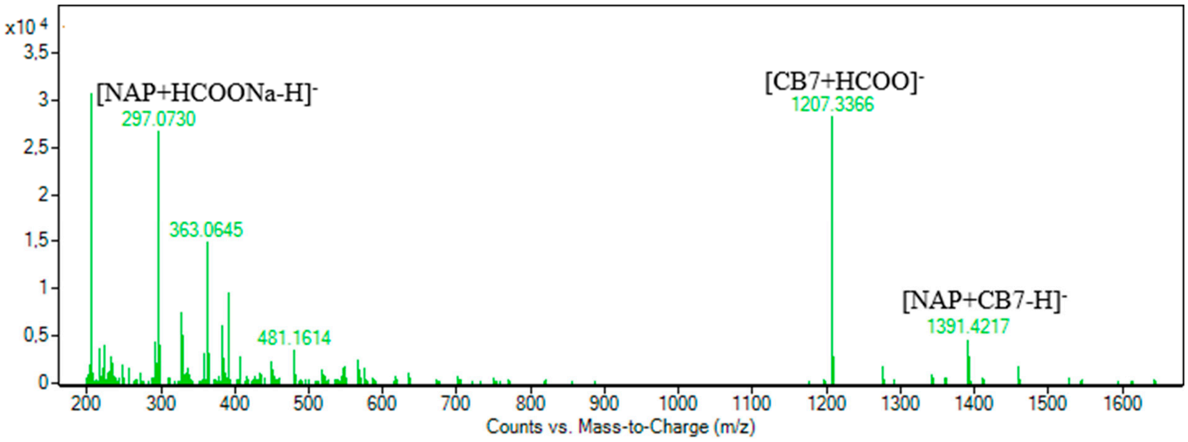

Figure S12. ESI– HRMS spectrum of naproxen and cucurbituril7 solution in molar ratio 1:1. c(NAP) = 2.15×10^{-5} M

2. NMR spectroscopy

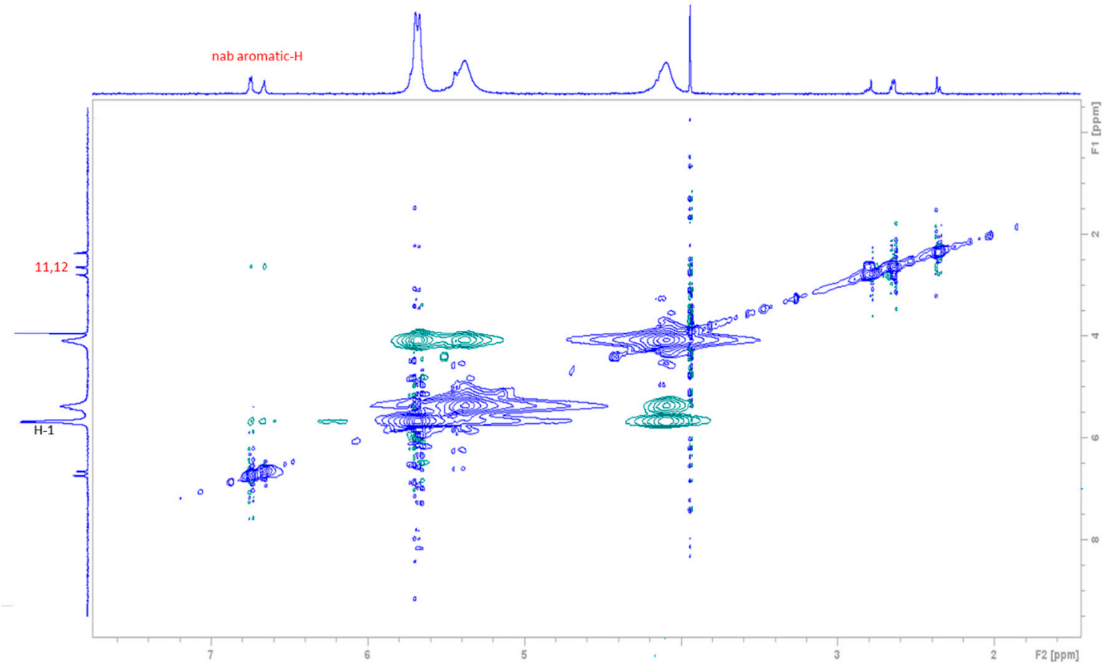

Figure S13. ROESY spectrum of NAB-CB7 1:1 complex in D_2O at 25 ºC.

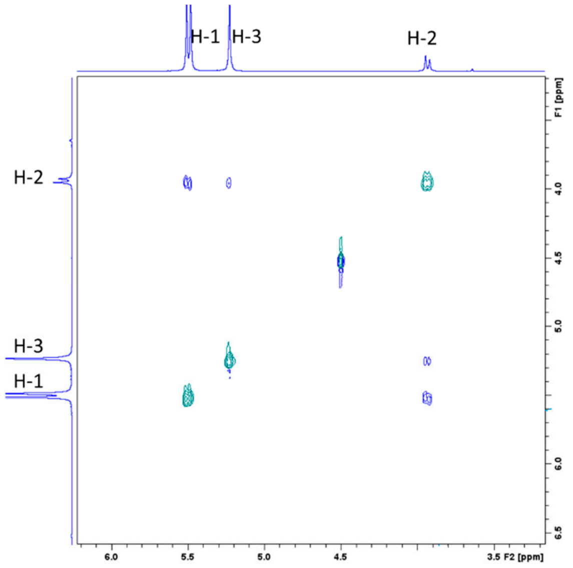

Figure S14. Part of the ROESY spectrum showing intramolecular interactions of CB7 in D_2O at 25 ºC.

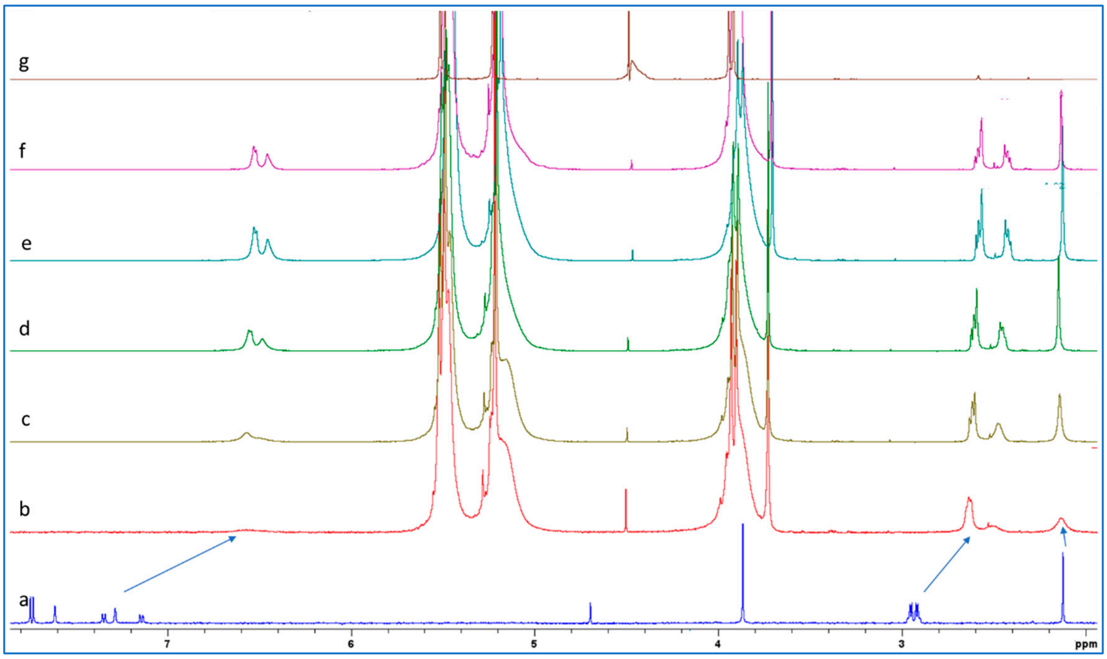

Figure S15. ^{1}H NMR spectra of (a) NAB (1.5 mM) (b) NAB (1.5 mM) - CB7 (0.5 mM) complex (c) NAB (1.5 mM) - CB7 (1.0 mM) complex (d) NAB (1.5 mM) - CB7 (1.5 mM) complex (e) NAB (1.5 mM) - CB7 (2.0 mM) complex (f) NAB (1.5 mM) - CB7 (2.5 mM) complex and (g) CB7 (10 mM) in D_2O at 25 °C.

Table S5. Comparision of ^{1}H NMR chemical shifts of protons 11 and 12 of NAB (1.5 mM), NAB (1.5 mM) - CB7 (0.5 mM) complex, NAB (1.5 mM) - CB7 (1.0 mM) complex, NAB (1.5 mM) - CB7 (1.5 mM) complex, NAB (1.5 mM) - CB7 (2.0 mM) complex and NAB (1.5 mM) - CB7 (2.5 mM) complex in D_2O at 25 °C.

[NAB] / M	[CB7] / M	Proton 11	Proton 12
1.5×10^{-3}	0	2.95	2.91
1.5×10^{-3}	0.0005	2.82	2.69
1.5×10^{-3}	0.001	2.81	2.68
1.5×10^{-3}	0.0015	2.8	2.67
1.5×10^{-3}	0.002	2.79	2.66
1.5×10^{-3}	0.0025	2.79	2.66

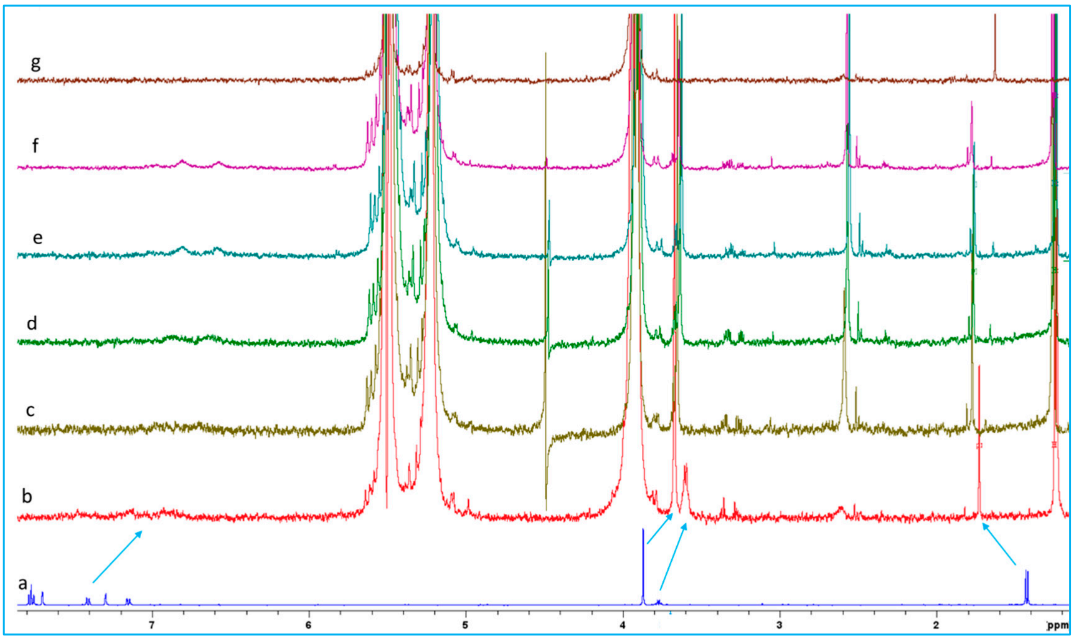

Figure S16. ^{1}H NMR spectra of (a) NAP (1.5 mM) (b) NAP (1.5 mM) - CB7 (0.5 mM) complex (c) NAP (1.5 mM) - CB7 (1.0 mM) complex (d) NAP (1.5 mM) - CB7 (1.5 mM) complex (e) NAP (1.5 mM) - CB7 (2.0 mM) complex (f) NAP (1.5 mM) - CB7 (2.5 mM) complex and (g) CB7 (10 mM) in D_2O at 25 °C.

Table S6. Comparision of ^{1}H NMR chemical shifts of protons 11 and 12 of NAP (1.5 mM), NAP (1.5 mM) - CB7 (0.5 mM) complex, NAP (1.5 mM) - CB7 (1.0 mM) complex, NAP (1.5 mM) - CB7 (1.5 mM) complex, NAP (1.5 mM) - CB7 (2.0 mM) complex and NAP (1.5 mM) - CB7 (2.5 mM) complex in D_2O at 25 °C.

[NAP] / M	[CB7] / M	Proton 11	Proton 13	Proton 14
1.5×10^{-3}	0	3.77	1.43	3.88
1.5×10^{-3}	0.0005	3.58	1.22	3.66
1.5×10^{-3}	0.001	3.68	1.26	3.66
1.5×10^{-3}	0.0015	3.68	1.26	3.66
1.5×10^{-3}	0.002	3.69	1.27	3.65
1.5×10^{-3}	0.0025	3.69	1.27	3.65

3. Isothermal titration calorimetry

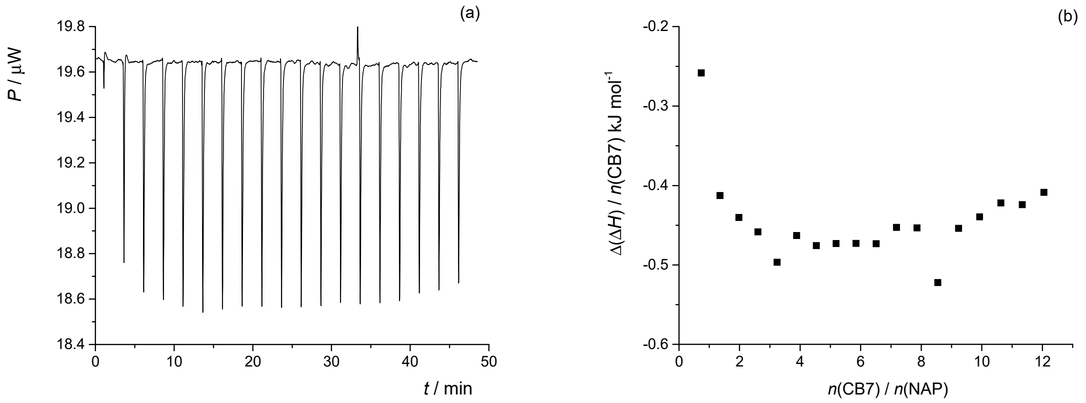

Figure S17. (a) Microcalorimetric titration of NAP (c_0 = 18.3 × 10^{-5} mol L^{-1}) with CB7 (c_0 = 11.4 × 10^{-3} mol L^{-1}) at pH 6.8 (phosphate buffer) at 298 K. (b) Dependence of successive enthalpy changes on CB7/NAP molar ratio. ●

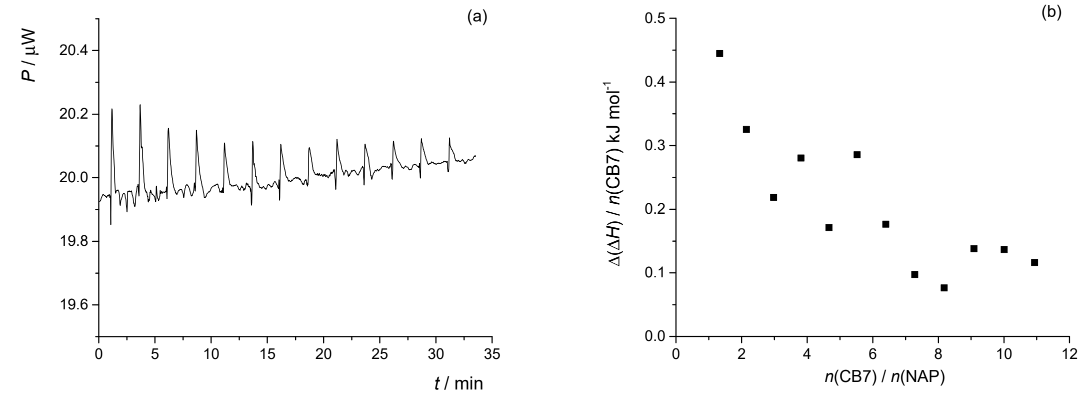

Figure S18. (a) Microcalorimetric titration of NAP (c_0 = 6.3 × 10^{-5} mol L^{-1}) with CB7 (c_0 = 3.4 × 10^{-3} mol L^{-1}) at pH 2 (HCl) at 298 K. (b) Dependence of successive enthalpy changes on CB7/NAP molar ratio. ●

Table S7. Equilibrium constant (log *K*) and thermodynamic parameters for complexation of NAB with CB7 at 25 °C in water.

Exp. No.	K_d (M)	ΔH (kJ/mol)	ΔG (kJ/mol)	$-T\Delta S$ (kJ/mol)	K_a (M^{-1})	Log*K*a
1	$(2.24\pm0.25)\times10^{-5}$	-21.3 ± 0.967	-26.6	-5.23	44642.86	4.6498
2	$(2.14\pm0.25)\times10^{-5}$	-19.7±0.932	-26.7	-7.01	46728.97	4.6696
3	$(2.15\pm0.33)\times10^{-5}$	-19.9±1.23	-26.7	-6.75	46511.63	4.6676
**Mean**		**-20.3 ± 0.9**	**-26.7 ± 0.1**	**-6.3 ± 0.9**		**4.66 ± 0.01**

3. Computational details

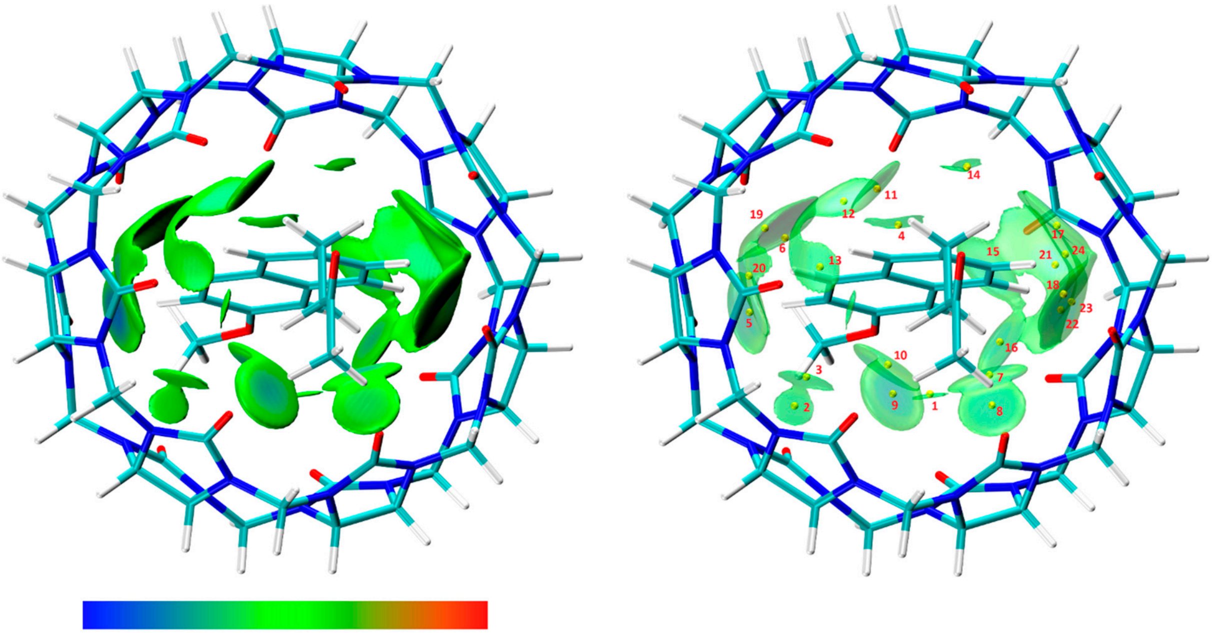

Figure S19. The structure and IGMH analysis (left) of NAB-CB7 1:1 host-guest complex. The isosurfaces of $\delta g^{inter} = 0.002$ a.u. are colored with $sign(\lambda_2)\rho$ in range from -0.05 (blue), 0.00 (green) and 0.05 (red), revealing weak attractive, van der Waals and repulsive interactions, respectively. The (3,-1) bond critical points (BCP, right) obtained by atoms-in-molecules (AIM) analysis of NAB-CB7 1:1 host-guest complex.

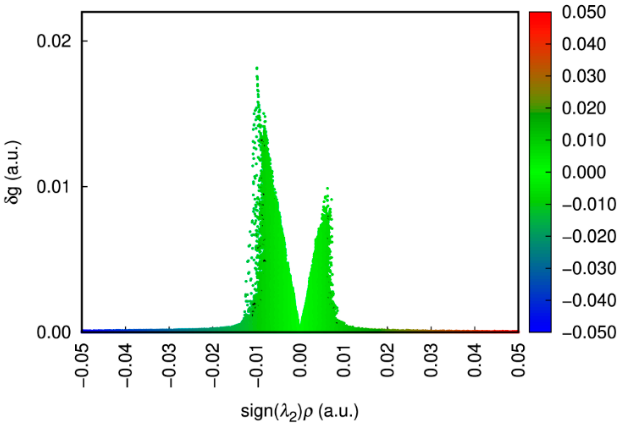

Figure S20. The fingerprint plot obtained by IGMH analysis of NAB-CB7 1:1 host-guest complex.

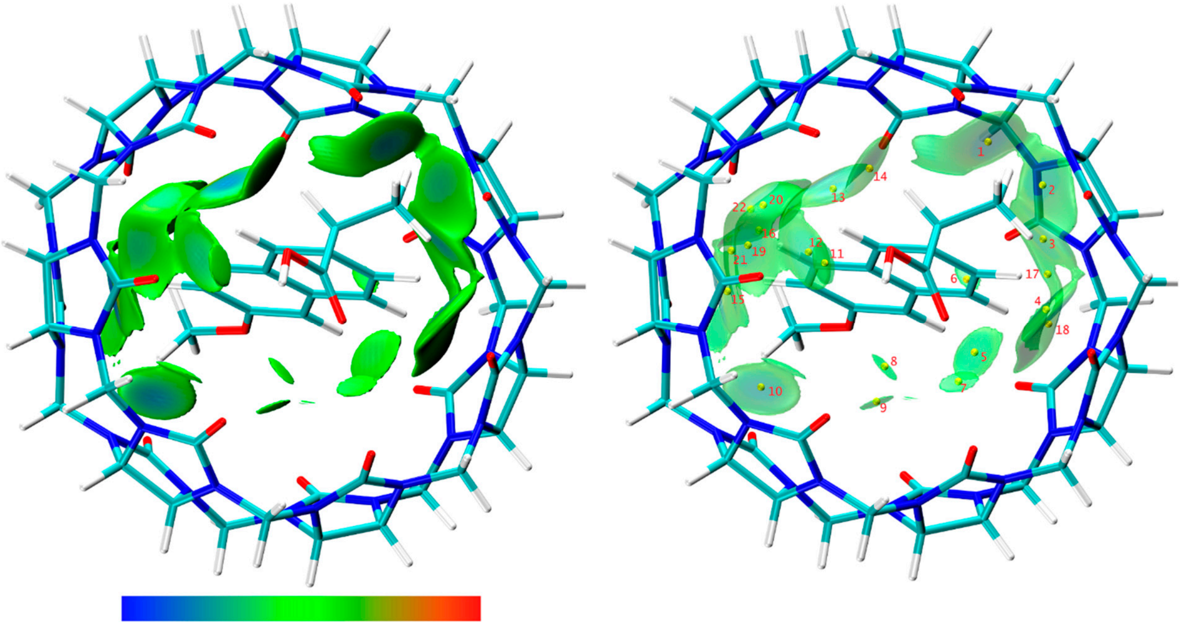

Figure S21. The structure and IGMH analysis (left) of NAP(acid)-CB7 1:1 host-guest complex. The isosurfaces of $\delta g^{inter} = 0.002$ a.u. are colored with $sign(\lambda_2)\rho$ in range from -0.05 (blue), 0.00 (green) and 0.05 (red), revealing weak attractive, van der Waals and repulsive interactions, respectively. The (3,-1) bond critical points (BCP, right) obtained by atoms-in-molecules (AIM) analysis of NAP(acid)-CB7 1:1 host-guest complex.

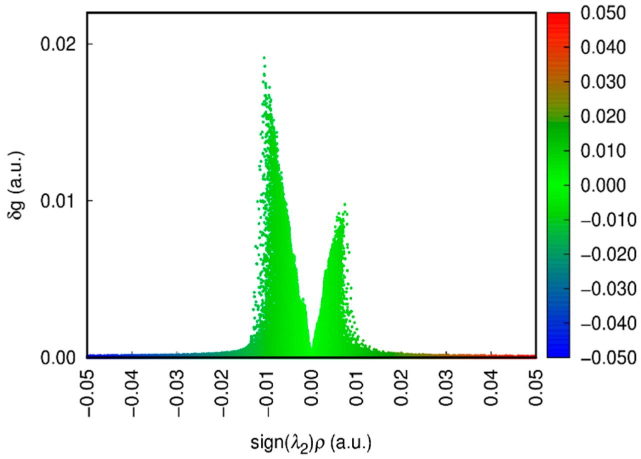

Figure S22. The fingerprint plot obtained by IGMH analysis of NAP(acid)-CB7 1:1 host-guest complex.

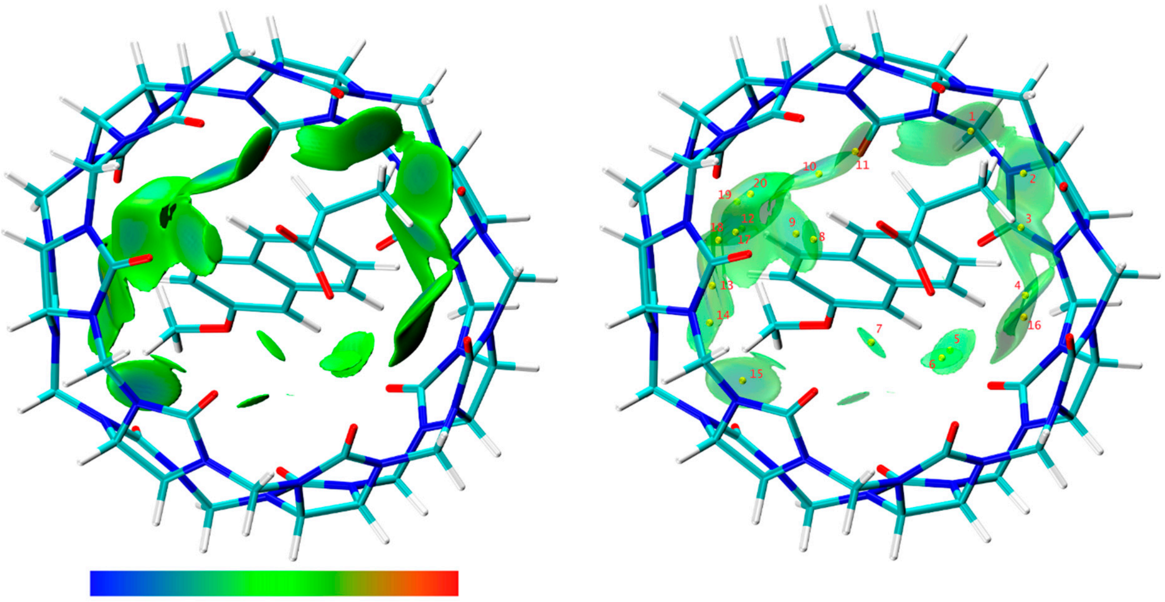

FigureS23. The structure and IGMH analysis (left) of NAP(anion)-CB7 1:1 host-guest complex. The isosurfaces of $\delta g^{inter} = 0.002$ a.u. are colored with *sign*(λ_2)ρ in range from -0.05 (blue), 0.00 (green) and 0.05 (red), revealing weak attractive, van der Waals and repulsive interactions, respectively. The (3,-1) bond critical points (BCP, right) obtained by atoms-in-molecules (AIM) analysis of NAP(anion)-CB7 1:1 host-guest complex.

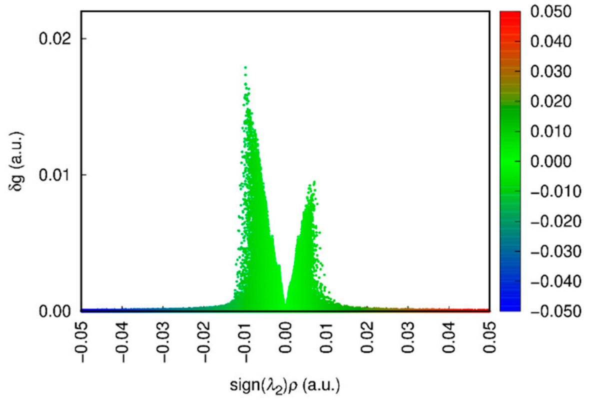

Figure S24. The fingerprint plot obtained by IGMH analysis of NPXan-CB7 1:1 host-guest complex.

Table S8. The values of density (ρ) and density gradient difference (δg) in *a.u.* for (3,-1) bond critical points (BCP) obtained by atoms-in-molecules (AIM) analysis of NAB-CB7 1:1 host-guest complex. The BCP points and the corresponding numbers are shown in Figure S19 (right).

BCP (3,-1)	ρ	δg	contact
1	0.002	0.003	O···Ar
2	0.002	0.004	O···H
3	0.002	0.004	O···H
4	0.002	0.003	O···H
5	0.010	0.019	O···H
6	0.004	0.008	O···H
7	0.008	0.014	O···H
8	0.005	0.009	O···H
9	0.008	0.016	O···H
10	0.008	0.015	O···H
11	0.005	0.009	O···H
12	0.004	0.007	O···H
13	0.003	0.005	O···H
14	0.002	0.003	O···H
15	0.005	0.010	O···H
16	0.007	0.013	O···H
17	0.008	0.016	O···H
18	0.008	0.016	O···H
19	0.005	0.009	N···H
20	0.007	0.013	N···H
21	0.005	0.009	N···H
22	0.006	0.011	N···H
23	0.005	0.009	N···H
24	0.004	0.008	N···H

Table S9. The values of density (ρ) and density gradient difference (δg) in *a.u.* for (3,-1) bond critical points (BCP) obtained by atoms-in-molecules (AIM) analysis of NAP(acid)-CB7 1:1 host-guest complex. The BCP points and the corresponding numbers are shown in Figure S21 (right).

BCP (3,-1)	ρ	δg	contact
1	0.009	0.018	O···H
2	0.007	0.012	O···H
3	0.008	0.017	O···H
4	0.008	0.016	O···H
5	0.004	0.007	O···H
6	0.002	0.003	O···H
7	0.002	0.003	O···H
8	0.001	0.002	O···H
9	0.002	0.003	O···H
10	0.008	0.015	O···H
11	0.002	0.003	O···H
12	0.005	0.009	O···H
13	0.009	0.017	O···H
14	0.006	0.011	O···H
15	0.011	0.020	O···H
16	0.006	0.010	O···H
17	0.004	0.006	N···H
18	0.007	0.011	N···H
19	0.005	0.009	N···H
20	0.004	0.008	N···H
21	0.005	0.010	N···H
22	0.005	0.010	N···H

Table S10. The values of density (ρ) and density gradient difference (δg) in *a.u.* for (3,-1) bond critical points (BCP) obtained by atoms-in-molecules (AIM) analysis of NAP(anion)-CB7 1:1 host-guest complex. The BCP points and the corresponding numbers are shown in Figure S23 (right).

BCP (3,-1)	ρ	δg	contact
1	0.007	0.013	O···H
2	0.006	0.012	O···H
3	0.008	0.015	O···H
4	0.008	0.016	O···H
5	0.003	0.006	O···H
6	0.002	0.004	O···H
7	0.002	0.004	O···H
8	0.002	0.005	O···H
9	0.005	0.010	O···H
10	0.009	0.017	O···H
11	0.005	0.009	O···H
12	0.006	0.011	O···H
13	0.010	0.019	O···H
14	0.004	0.006	O···H
15	0.007	0.014	O···H
16	0.007	0.014	N···H
17	0.006	0.011	N···H
18	0.005	0.009	N···H
19	0.005	0.009	N···H
20	0.005	0.010	N···H

Coordinates of the optimized structures of NAB, NAP(acid), NAP(anion), CB7, NAB-CB7, NAP(acid)-CB7 and NAP(anion)-CB7 host-guest inclusion complexes calculated at B3LYP-D3/6-311++G(2d,2p) (PCM) level with water as a solvent.

NAB:

C	3.750338	0.097863	0.176353
C	2.819199	1.112878	0.108970
C	1.458014	0.830760	-0.130861
C	1.041790	-0.524724	-0.306833
C	2.022546	-1.540088	-0.232618
C	3.344934	-1.247921	0.002858
H	0.773391	2.882292	-0.084315
H	3.144627	2.135750	0.242617
C	0.471445	1.850481	-0.209546
C	-0.327854	-0.804919	-0.548911
H	1.721309	-2.571174	-0.364484
H	4.064029	-2.049958	0.052999
C	-0.841660	1.539571	-0.444845
H	-0.627731	-1.837651	-0.681029
H	-1.576152	2.333086	-0.504657
C	-1.268009	0.194519	-0.619515
C	-2.729649	-0.105652	-0.844618
C	-3.544984	0.002633	0.446981
H	-3.142710	0.585955	-1.580582
H	-2.846332	-1.106978	-1.257998
H	-3.167541	-0.693869	1.201308
H	-3.436179	0.994655	0.895291
O	5.042768	0.472397	0.413213
C	6.048758	-0.539611	0.492793
H	6.130377	-1.092747	-0.444375
H	6.979735	-0.014605	0.682860
H	5.850698	-1.232715	1.312143
C	-5.027047	-0.259220	0.275615
O	-5.516616	-0.513037	-0.810043
C	-5.866719	-0.189295	1.527910
H	-5.517319	-0.930860	2.248565
H	-5.753350	0.788421	1.999127
H	-6.912680	-0.368137	1.294766

NAP(acid):

C	3.410039	0.218700	0.091708
C	2.392279	1.149484	0.109487
C	1.047969	0.752658	-0.043931
C	0.740159	-0.631541	-0.219145
C	1.806886	-1.559044	-0.232197
C	3.111654	-1.155271	-0.081528
H	0.192949	2.733878	0.105612
H	2.636655	2.194603	0.242841
C	-0.025976	1.682897	-0.030347
C	-0.612630	-1.027859	-0.371783
H	1.586877	-2.610315	-0.363910
H	3.899331	-1.891667	-0.096613
C	-1.320700	1.264811	-0.185365
H	-0.831578	-2.080533	-0.501299
H	-2.122450	1.990809	-0.167896
C	-1.633447	-0.109869	-0.361673
C	-3.080199	-0.544532	-0.555118
H	-3.099455	-1.633032	-0.592412
O	4.677018	0.701266	0.247617
C	5.771801	-0.218114	0.234237
H	5.835183	-0.745101	-0.719116
H	6.663591	0.385176	0.372189

H	5.693376	-0.939769	1.048876
C	-3.880955	-0.132983	0.668821
O	-4.567392	0.858834	0.758927
C	-3.698334	0.023761	-1.837060
H	-4.724598	-0.321369	-1.957750
H	-3.706612	1.111630	-1.818599
H	-3.117401	-0.303794	-2.697630
O	-3.717700	-0.998151	1.690330
H	-4.212556	-0.664704	2.454080

NAP(anion):

C	3.381820	0.208273	0.093084
C	2.367490	1.142307	0.115185
C	1.020704	0.752002	-0.039434
C	0.705966	-0.629424	-0.221726
C	1.770105	-1.559834	-0.238929
C	3.077903	-1.162899	-0.086240
H	0.171457	2.734347	0.121809
H	2.615908	2.186088	0.253609
C	-0.051660	1.684498	-0.020393
C	-0.650059	-1.017937	-0.379285
H	1.546335	-2.609978	-0.375449
H	3.862095	-1.903085	-0.104502
C	-1.348041	1.270966	-0.175050
H	-0.870687	-2.069563	-0.518447
H	-2.152265	1.991900	-0.140857
C	-1.675190	-0.101777	-0.361165
C	-3.119267	-0.529796	-0.526843
H	-3.129122	-1.612216	-0.650468
O	4.653278	0.686489	0.251680
C	5.742026	-0.238223	0.233508
H	5.802118	-0.762663	-0.721738
H	6.638053	0.358742	0.372875
H	5.661046	-0.963398	1.045094
C	-3.903442	-0.225887	0.787085
O	-4.375746	0.930912	0.924776
C	-3.773165	0.114438	-1.756113
H	-4.799850	-0.235821	-1.871715
H	-3.801252	1.197792	-1.658369
H	-3.220855	-0.139233	-2.662580
O	-3.976797	-1.169044	1.619623

CB7:

N	2.320083	4.763028	-1.195524
C	2.996156	5.034652	0.053843
C	4.217469	4.069379	0.023187
N	2.284195	4.634774	1.250800
N	4.031488	3.334744	-1.211783
N	4.068665	3.297758	1.237222
C	5.048593	2.485899	-1.788596
C	1.257876	5.585798	-1.726902
C	2.962814	3.802896	-1.946128
C	5.101818	2.442218	1.773379
C	1.213850	5.412707	1.830735
C	2.958827	3.667564	1.965219
O	2.658958	3.451121	-3.073925
O	2.644713	3.237656	3.062848
N	5.092044	1.148882	-1.244428
N	5.138757	1.114549	1.204143
C	5.811676	0.798939	-0.039271
C	4.698990	0.042520	-1.965533
C	4.838374	-0.011205	1.940906
O	4.376748	-0.027770	3.069923
N	5.184742	-1.114118	1.191227

O	4.151320	0.054537	-3.055656
N	5.065015	-1.080519	-1.254835
C	5.817865	-0.758318	-0.060076
C	5.172417	-2.454897	1.729433
C	5.016949	-2.405512	-1.828688
N	4.021040	-3.274547	-1.244779
N	4.132117	-3.307478	1.202081
N	-0.054610	5.293959	-1.197902
N	-0.085374	5.167287	1.248230
C	4.247799	-4.043400	-0.036969
C	3.040653	-3.695204	1.947883
C	2.934966	-3.730489	-1.961316
O	2.756343	-3.294643	3.064400
N	2.346021	-4.641558	1.224514
O	2.593214	-3.346274	-3.067577
N	2.324468	-4.720842	-1.223410
C	3.031351	-5.014850	0.003000
C	-0.554528	5.830934	0.048247
C	-1.040494	4.687680	-1.945524
C	-1.111672	4.586430	1.962730
O	-1.014999	4.065544	3.061655
N	-2.271450	4.729219	1.232951
O	-0.910160	4.228120	-3.067975
N	-2.209634	4.723200	-1.215543
C	-2.070726	5.479064	0.013187
N	-4.169022	3.208765	1.199011
N	-4.105998	3.204364	-1.249531
C	-4.378512	2.065292	1.940010
C	-4.909087	3.204565	-0.046146
C	-4.230933	2.035385	-1.968155
O	-3.733475	1.807344	-3.058556
N	-5.045440	1.182469	-1.254618
O	-3.970625	1.858431	3.070878
N	-5.159851	1.211913	1.191842
C	-5.573703	-0.035868	-1.823244
C	-5.582962	1.800625	-0.060324
C	-5.721074	-0.003925	1.734252
N	-5.043757	-1.246568	-1.239268
N	-5.147099	-1.220634	1.207958
C	-4.263272	-2.126765	-1.958251
C	-5.572100	-1.840370	-0.027008
C	-4.319558	-2.033912	1.950692
O	-3.883278	-1.788549	3.063046
N	-4.100129	-3.188973	1.229999
O	-3.797271	-1.929694	-3.068302
N	-4.132259	-3.280918	-1.217513
C	-3.481945	-4.355501	1.817895
C	-3.521569	-4.479774	-1.743082
C	-4.888044	-3.238517	0.014256
N	-2.222294	-4.787334	-1.189826
N	-2.195214	-4.696457	1.257839
C	-1.066125	-4.717561	-1.936847
C	-2.032531	-5.492876	0.060016
C	-1.023700	-4.572995	1.972894
O	-0.902122	-4.039953	3.063567
N	-0.020986	-5.188505	1.255496
O	-0.969567	-4.275354	-3.069560
N	-0.045158	-5.268080	-1.192395
C	-0.512422	-5.832778	0.056286
C	-3.572605	4.397247	1.764881
C	-3.489454	4.389649	-1.797660
C	1.259287	-5.541179	-1.751308
C	1.289328	-5.433844	1.811072
H	3.274014	6.087469	0.112808

H	5.179206	4.583380	0.016769
H	4.841263	2.403652	-2.850839
H	6.024350	2.953791	-1.646705
H	1.491230	6.632850	-1.526579
H	1.223164	5.423674	-2.799517
H	4.921573	2.335729	2.838444
H	6.069594	2.921976	1.617401
H	1.155176	5.157447	2.884071
H	1.452550	6.473173	1.729514
H	6.809231	1.239333	-0.052504
H	6.817877	-1.189647	-0.114675
H	5.020131	-2.375734	2.801141
H	6.139508	-2.921368	1.535020
H	4.781115	-2.299245	-2.882836
H	5.998828	-2.870737	-1.724204
H	5.211077	-4.552081	-0.087023
H	3.314287	-6.067578	0.033896
H	-0.356652	6.901725	0.105678
H	-2.721365	6.353880	-0.005041
H	-5.621220	4.030208	-0.066130
H	-6.659582	-0.034246	-1.712447
H	-5.321012	-0.043664	-2.878815
H	-6.671513	1.840091	-0.111578
H	-6.795287	-0.012561	1.542749
H	-5.546755	0.005218	2.805485
H	-6.660640	-1.888329	-0.071795
H	-4.153079	-5.208569	1.702997
H	-3.337851	-4.152420	2.874385
H	-4.191246	-5.322080	-1.561664
H	-3.397138	-4.343529	-2.812678
H	-5.594142	-4.068526	0.054084
H	-2.676031	-6.372346	0.099878
H	-0.304554	-6.902772	0.089210
H	-4.243346	5.242652	1.601759
H	-3.459166	4.229555	2.831253
H	-4.170096	5.232543	-1.665295
H	-3.331663	4.219178	-2.857937
H	1.201627	-5.357893	-2.819580
H	1.506059	-6.589961	-1.577614
H	1.248955	-5.193820	2.868861
H	1.530345	-6.491431	1.690363

NAB-CB7:

N	5.129086	1.353180	1.188954
C	5.610157	2.010527	-0.011610
C	4.850277	3.369104	-0.002212
N	5.207748	1.410623	-1.262542
N	4.125676	3.342177	1.248142
N	4.037015	3.304346	-1.201620
C	3.471940	4.495955	1.820064
C	5.711278	0.140943	1.717694
C	4.339100	2.181574	1.958078
C	3.351985	4.449393	-1.753517
C	5.823296	0.234534	-1.827082
C	4.323654	2.196040	-1.969303
O	3.926672	1.938802	3.079681
O	3.895621	1.965973	-3.087122
N	2.152894	4.761770	1.294209
N	2.067345	4.715485	-1.152314
C	1.913260	5.485256	0.063840
C	1.011005	4.618159	2.052060
C	0.887920	4.598715	-1.856558
O	0.757208	4.092873	-2.958592
N	-0.111535	5.187669	-1.112443

O	0.947762	4.139204	3.172344
N	-0.040625	5.146122	1.335229
C	0.386711	5.784377	0.107877
C	-1.420257	5.461853	-1.659486
C	-1.348126	5.345445	1.911328
N	-2.386622	4.530989	1.324553
N	-2.475954	4.635914	-1.122172
N	5.198463	-1.070702	1.119933
N	5.289921	-1.011265	-1.327844
C	-3.127004	4.912319	0.141499
C	-3.192949	3.754523	-1.903353
C	-2.992321	3.504446	2.015688
O	-2.959678	3.472506	-3.065682
N	-4.240847	3.274082	-1.144499
O	-2.631746	3.048478	3.087760
N	-4.098460	3.106595	1.297105
C	-4.339795	3.938081	0.138522
C	5.727749	-1.642042	-0.101332
C	4.398867	-1.955076	1.811796
C	4.515100	-1.843693	-2.104985
O	4.098513	-1.596855	-3.224044
N	4.318185	-3.013141	-1.405300
O	3.921055	-1.773283	2.919215
N	4.258457	-3.090035	1.044209
C	5.059208	-3.047660	-0.161422
N	2.423993	-4.527608	-1.498059
N	2.356645	-4.597428	0.948825
C	1.264542	-4.357696	-2.221796
C	2.225085	-5.315939	-0.304091
C	1.186275	-4.599510	1.679265
O	1.047824	-4.177909	2.815800
N	0.210026	-5.194502	0.910253
O	1.166769	-3.797322	-3.300741
N	0.238334	-4.965293	-1.528093
C	-1.097108	-5.526082	1.422274
C	0.712899	-5.675765	-0.355804
C	-1.040371	-5.233521	-2.146244
N	-2.171294	-4.716728	0.892464
N	-2.149832	-4.530266	-1.549327
C	-2.902505	-3.864572	1.691352
C	-2.835367	-4.996812	-0.365020
C	-2.847902	-3.545236	-2.214072
O	-2.549152	-3.056967	-3.290989
N	-3.963649	-3.234728	-1.462781
O	-2.632836	-3.551368	2.839332
N	-4.007379	-3.459917	0.978167
C	-5.047620	-2.435574	-1.991008
C	-5.059661	-2.662926	1.556500
C	-4.109044	-4.103847	-0.311759
N	-5.115641	-1.305821	1.065982
N	-5.180431	-1.133910	-1.379683
C	-4.731867	-0.232370	1.838376
C	-5.850025	-0.906514	-0.114084
C	-4.947939	0.034473	-2.070175
O	-4.528846	0.124151	-3.212260
N	-5.296819	1.087210	-1.251349
O	-4.172249	-0.289041	2.920462
N	-5.122593	0.917847	1.189136
C	-5.892537	0.646834	-0.007401
C	3.725258	-4.180075	-2.015087
C	3.635533	-4.291624	1.548325
C	-5.085988	2.206492	1.838046
C	-5.298149	2.460763	-1.703044
H	6.694836	2.116410	0.024979

H	5.509881	4.236900	-0.039109
H	3.369524	4.320007	2.886211
H	4.100296	5.373015	1.656617
H	6.793122	0.171907	1.577280
H	5.486617	0.106256	2.779189
H	3.189374	4.255423	-2.809091
H	3.984504	5.332746	-1.642141
H	5.652492	0.255963	-2.898863
H	6.895722	0.267428	-1.629979
H	2.532661	6.382077	0.025851
H	0.151571	6.849225	0.132230
H	-1.378383	5.280493	-2.728905
H	-1.660245	6.511782	-1.479913
H	-1.283025	5.092569	2.964927
H	-1.626520	6.395844	1.808038
H	-3.414441	5.963169	0.197897
H	-5.303491	4.440696	0.228582
H	6.816853	-1.677202	-0.061051
H	5.774717	-3.870627	-0.169546
H	2.881013	-6.187120	-0.311014
H	-1.302786	-6.575408	1.202356
H	-1.076443	-5.379575	2.497671
H	0.522472	-6.744817	-0.457767
H	-1.238340	-6.306409	-2.099685
H	-0.975260	-4.924580	-3.184698
H	-3.054128	-6.062028	-0.447892
H	-5.982112	-2.985910	-1.867906
H	-4.860070	-2.279015	-3.048779
H	-6.019119	-3.145130	1.362709
H	-4.885945	-2.621901	2.627287
H	-5.040109	-4.667519	-0.380674
H	-6.836697	-1.370821	-0.119915
H	-6.900734	1.050317	0.091428
H	4.395465	-5.030481	-1.878801
H	3.615681	-3.974288	-3.075188
H	4.312584	-5.133216	1.388575
H	3.474406	-4.156265	2.613188
H	-4.845657	2.042827	2.883888
H	-6.070465	2.671461	1.759914
H	-5.170024	2.450890	-2.780914
H	-6.260659	2.911692	-1.455581
C	-0.000783	0.187221	-3.199810
C	1.042458	0.107431	-2.303161
C	0.809425	0.264190	-0.920803
C	-0.517405	0.525150	-0.455586
C	-1.552460	0.631761	-1.409350
C	-1.313696	0.459506	-2.749903
H	2.858444	-0.031704	-0.291830
H	2.041792	-0.091691	-2.664691
C	1.844385	0.139471	0.043462
C	-0.762670	0.615148	0.936913
H	-2.555518	0.843513	-1.066876
H	-2.140403	0.526433	-3.436091
C	1.565904	0.212305	1.382810
H	-1.776318	0.792383	1.276364
H	2.360824	0.087871	2.103953
C	0.244038	0.435519	1.853741
C	-0.052135	0.352612	3.329481
C	-0.287845	-1.106867	3.732386
H	0.779464	0.757844	3.906893
H	-0.931724	0.948822	3.570530
H	-1.140077	-1.528568	3.191000
H	0.555735	-1.738077	3.438103
O	0.312669	-0.005849	-4.520596

C	-0.755078	-0.057878	-5.467378
H	-1.279439	0.897441	-5.530822
H	-0.291514	-0.276609	-6.424855
H	-1.465559	-0.847845	-5.217204
C	-0.533643	-1.339902	5.202728
O	-0.540192	-0.432044	6.018369
C	-0.779030	-2.772871	5.606222
H	-1.670245	-3.140630	5.095414
H	0.046898	-3.398549	5.264398
H	-0.897572	-2.853266	6.683505

NAP(acid)-CB7:

N	-4.744421	-2.393098	0.956439
C	-5.059161	-3.060088	-0.291899
C	-4.017771	-4.215456	-0.355543
N	-4.794298	-2.306027	-1.495234
N	-3.290697	-4.085319	0.888275
N	-3.264276	-3.921889	-1.557128
C	-2.398113	-5.103697	1.393025
C	-5.594333	-1.384072	1.548947
C	-3.792916	-3.077908	1.686503
C	-2.368013	-4.866568	-2.180007
C	-5.655753	-1.263539	-1.994663
C	-3.773018	-2.842953	-2.245920
O	-3.473314	-2.852490	2.839720
O	-3.405038	-2.453356	-3.341586
N	-1.062818	-5.058977	0.837173
N	-1.045704	-4.894660	-1.606334
C	-0.710103	-5.666144	-0.431395
C	0.046533	-4.789604	1.612192
C	0.066255	-4.450233	-2.286916
O	0.066306	-3.843079	-3.345113
N	1.181108	-4.843152	-1.578020
O	0.037007	-4.405939	2.769036
N	1.168430	-5.065802	0.859897
C	0.846060	-5.649797	-0.423114
C	2.508039	-4.786467	-2.143856
C	2.500199	-5.065715	1.417998
N	3.355293	-4.008077	0.928250
N	3.377896	-3.820031	-1.516763
N	-5.372506	-0.046881	1.042666
N	-5.416877	0.032926	-1.408376
C	4.115410	-4.093977	-0.300220
C	3.877829	-2.734500	-2.203145
C	3.754286	-2.955043	1.720646
O	3.542407	-2.372224	-3.317468
N	4.849237	-2.149108	-1.418669
O	3.350549	-2.708605	2.845294
N	4.708933	-2.241639	1.027752
C	5.102362	-2.894124	-0.205110
C	-5.997465	0.471237	-0.158616
C	-4.803978	0.949376	1.809147
C	-4.838154	1.061619	-2.116311
O	-4.366279	0.983258	-3.238112
N	-4.914177	2.201592	-1.347244
O	-4.325803	0.811862	2.922346
N	-4.893443	2.129627	1.103892
C	-5.654292	1.990884	-0.119456
N	-3.411778	4.108666	-1.364526
N	-3.324403	3.984007	1.083043
C	-2.238925	4.198729	-2.080975
C	-3.355135	4.815220	-0.107721
C	-2.186778	4.202700	1.835241

O	-1.970694	3.760666	2.950360
N	-1.356385	5.036826	1.119636
O	-2.041740	3.752129	-3.198756
N	-1.335588	4.917377	-1.324371
C	-0.154997	5.613642	1.676241
C	-1.943295	5.466908	-0.126291
C	-0.130137	5.464691	-1.903790
N	1.076444	5.087250	1.134543
N	1.089218	4.968061	-1.311667
C	1.940479	4.313816	1.878767
C	1.664892	5.534532	-0.108682
C	1.999548	4.220523	-2.029241
O	1.814074	3.729654	-3.130223
N	3.167185	4.156755	-1.299287
O	1.731320	3.879792	2.999188
N	3.097421	4.149188	1.148567
C	4.399927	3.638506	-1.847216
C	4.288489	3.562542	1.713454
C	3.096510	4.923896	-0.075291
N	4.664919	2.297745	1.125334
N	4.793586	2.350872	-1.323215
C	4.551772	1.109155	1.811335
C	5.484071	2.177233	-0.060603
C	4.837274	1.219270	-2.106326
O	4.457086	1.128447	-3.262141
N	5.412192	0.212288	-1.362463
O	4.005303	0.950791	2.889084
N	5.191457	0.131105	1.078337
C	5.883913	0.671687	-0.074531
C	-4.611447	3.506675	-1.890347
C	-4.515246	3.403688	1.666927
C	5.469368	-1.174640	1.632846
C	5.705459	-1.092281	-1.907891
H	-6.093668	-3.406093	-0.283846
H	-4.470501	-5.205438	-0.426320
H	-2.310993	-4.960435	2.465608
H	-2.831810	-6.085590	1.191509
H	-6.638778	-1.654288	1.384801
H	-5.389882	-1.369429	2.615072
H	-2.270172	-4.585892	-3.223998
H	-2.801430	-5.866265	-2.112947
H	-5.482236	-1.176717	-3.062716
H	-6.694376	-1.547086	-1.819209
H	-1.135376	-6.668051	-0.501867
H	1.293459	-6.640514	-0.511661
H	2.411455	-4.509127	-3.188876
H	2.963730	-5.776027	-2.073012
H	2.405102	-4.940841	2.492057
H	2.973336	-6.026008	1.204547
H	4.612109	-5.062571	-0.368630
H	6.149973	-3.193461	-0.156591
H	-7.068132	0.264746	-0.145710
H	-6.533410	2.635477	-0.093344
H	-4.168678	5.538672	-0.042177
H	-0.178534	6.692611	1.512708
H	-0.156193	5.410183	2.742499
H	-1.966772	6.556155	-0.179456
H	-0.150572	6.552742	-1.807485
H	-0.120254	5.197545	-2.955718
H	1.656386	6.623946	-0.160997
H	5.196588	4.360125	-1.656770
H	4.265271	3.524430	-2.918265
H	5.119675	4.261459	1.603897
H	4.099087	3.392860	2.768707

H	3.889677	5.672071	-0.051629
H	6.337344	2.853615	-0.001327
H	6.957590	0.504263	0.016423
H	-5.453198	4.173905	-1.698234
H	-4.479125	3.392557	-2.961607
H	-5.345634	4.102949	1.551118
H	-4.321034	3.249504	2.723687
H	5.214084	-1.148353	2.687634
H	6.534437	-1.385483	1.522615
H	5.572516	-1.031733	-2.983592
H	6.742214	-1.346870	-1.684039
C	0.192361	0.250996	-2.438465
C	-0.853890	0.168570	-1.545257
C	-0.612379	-0.119854	-0.184211
C	0.729319	-0.322755	0.265220
C	1.775432	-0.232777	-0.682080
C	1.524449	0.045734	-2.001931
H	-2.675760	-0.084898	0.441699
H	-1.864463	0.327212	-1.898227
C	-1.655321	-0.222453	0.770228
C	0.966130	-0.622117	1.627793
H	2.794079	-0.395204	-0.356412
H	2.344510	0.097747	-2.697414
C	-1.390709	-0.511948	2.084358
H	1.984449	-0.778850	1.956045
H	-2.216691	-0.594665	2.770872
C	-0.063730	-0.726379	2.533742
C	0.256932	-1.087623	3.976653
H	1.153522	-1.704108	3.968553
O	-0.130929	0.525316	-3.737517
C	0.934489	0.722852	-4.673081
H	1.523309	-0.187357	-4.797885
H	0.453947	0.972048	-5.614686
H	1.579745	1.542778	-4.359182
C	0.560874	0.150663	4.798797
O	-0.125877	1.145284	4.852961
C	-0.859114	-1.858297	4.707617
H	-0.510964	-2.179575	5.689562
H	-1.741482	-1.236560	4.845843
H	-1.137880	-2.731105	4.126228
O	1.682752	0.012027	5.539860
H	1.794795	0.817356	6.067662

NAP(anion)-CB7:

N	-4.755019	-2.383609	0.935228
C	-5.067599	-3.057803	-0.309354
C	-4.025453	-4.212806	-0.365825
N	-4.802900	-2.311137	-1.517526
N	-3.295188	-4.072291	0.874959
N	-3.275879	-3.929323	-1.571795
C	-2.405118	-5.090889	1.385122
C	-5.610249	-1.375802	1.522890
C	-3.803344	-3.064165	1.670272
C	-2.376694	-4.875747	-2.186574
C	-5.661960	-1.268547	-2.020261
C	-3.784352	-2.854704	-2.266815
O	-3.489753	-2.836228	2.823725
O	-3.418529	-2.474415	-3.366388
N	-1.068228	-5.049991	0.832479
N	-1.054550	-4.894422	-1.611962
C	-0.715398	-5.660100	-0.433957
C	0.041425	-4.777297	1.607231
C	0.055554	-4.453406	-2.297533

O	0.052709	-3.854164	-3.360163
N	1.172091	-4.838921	-1.587516
O	0.031044	-4.395068	2.763720
N	1.164008	-5.048981	0.852237
C	0.840673	-5.639827	-0.427444
C	2.497568	-4.784251	-2.156535
C	2.496875	-5.049987	1.409133
N	3.353453	-3.995528	0.913848
N	3.369520	-3.817975	-1.532793
N	-5.389794	-0.038417	1.016021
N	-5.420286	0.028959	-1.437863
C	4.108648	-4.090592	-0.317435
C	3.864766	-2.730288	-2.218781
C	3.784631	-2.959869	1.714533
O	3.523723	-2.365616	-3.330738
N	4.839459	-2.146190	-1.437822
O	3.410623	-2.727141	2.851063
N	4.734404	-2.248762	1.011270
C	5.104424	-2.897253	-0.230242
C	-6.004775	0.474801	-0.192766
C	-4.834341	0.963015	1.786087
C	-4.834527	1.052247	-2.147702
O	-4.362738	0.967966	-3.269058
N	-4.905350	2.195166	-1.382083
O	-4.377849	0.835027	2.909122
N	-4.908780	2.138139	1.070011
C	-5.657163	1.993539	-0.159740
N	-3.403368	4.102805	-1.401539
N	-3.332856	3.988070	1.045845
C	-2.227909	4.193906	-2.113738
C	-3.353962	4.815231	-0.147189
C	-2.190687	4.190654	1.795616
O	-1.975262	3.740530	2.907100
N	-1.352822	5.019810	1.081913
O	-2.021791	3.737670	-3.225980
N	-1.335200	4.928135	-1.362605
C	-0.161180	5.603463	1.653972
C	-1.940246	5.465402	-0.159077
C	-0.114266	5.450180	-1.927328
N	1.079580	5.087630	1.125945
N	1.089038	4.932411	-1.317269
C	1.935158	4.304297	1.869222
C	1.665523	5.517966	-0.122340
C	2.013200	4.205118	-2.040492
O	1.836492	3.719048	-3.144631
N	3.181203	4.155760	-1.310858
O	1.721424	3.872751	2.989173
N	3.089892	4.127672	1.136690
C	4.419460	3.647055	-1.855614
C	4.280157	3.541407	1.703170
C	3.097808	4.911714	-0.081507
N	4.662268	2.281987	1.106632
N	4.818908	2.358641	-1.340116
C	4.571083	1.093418	1.797571
C	5.494792	2.174254	-0.072118
C	4.842792	1.225540	-2.120966
O	4.457090	1.137842	-3.275108
N	5.407117	0.212220	-1.377893
O	4.030082	0.931897	2.876950
N	5.225681	0.123950	1.066296
C	5.897668	0.669175	-0.095163
C	-4.599620	3.497544	-1.930645
C	-4.528392	3.414256	1.626867
C	5.506704	-1.185864	1.609753

C	5.694473	-1.090360	-1.930187
H	-6.101831	-3.404910	-0.300415
H	-4.478001	-5.203620	-0.427180
H	-2.321365	-4.946882	2.457679
H	-2.839759	-6.072729	1.184583
H	-6.653273	-1.649866	1.355040
H	-5.410427	-1.358662	2.589910
H	-2.279441	-4.603076	-3.232782
H	-2.806633	-5.876437	-2.111991
H	-5.488182	-1.185776	-3.088615
H	-6.701348	-1.549056	-1.844071
H	-1.137823	-6.663568	-0.500640
H	1.289864	-6.630213	-0.511990
H	2.398419	-4.508469	-3.201748
H	2.952829	-5.774064	-2.085285
H	2.403889	-4.919901	2.482825
H	2.966924	-6.012914	1.199522
H	4.599155	-5.062652	-0.383602
H	6.150661	-3.204038	-0.198840
H	-7.076368	0.272276	-0.184808
H	-6.535195	2.640262	-0.146426
H	-4.167025	5.539963	-0.090235
H	-0.190527	6.682747	1.493176
H	-0.172451	5.396963	2.719656
H	-1.961512	6.555255	-0.199661
H	-0.116066	6.538877	-1.834884
H	-0.096147	5.179386	-2.978183
H	1.655075	6.606557	-0.191133
H	5.210917	4.371447	-1.655503
H	4.290836	3.540336	-2.928184
H	5.109823	4.244012	1.603385
H	4.086255	3.362294	2.756097
H	3.888534	5.662080	-0.045072
H	6.346249	2.851456	0.003075
H	6.973126	0.504291	-0.021787
H	-5.442724	4.165788	-1.747151
H	-4.461211	3.377546	-3.000531
H	-5.354990	4.116640	1.502889
H	-4.340279	3.264473	2.685366
H	5.266286	-1.164374	2.668157
H	6.569459	-1.398618	1.483307
H	5.553452	-1.025759	-3.004697
H	6.732646	-1.348137	-1.715639
C	0.233940	0.237251	-2.306537
C	-0.815581	0.196217	-1.414918
C	-0.586222	-0.080077	-0.048042
C	0.747719	-0.310780	0.411584
C	1.797849	-0.258444	-0.534569
C	1.558879	0.007158	-1.860330
H	-2.649622	0.002393	0.568061
H	-1.821713	0.373510	-1.772551
C	-1.633645	-0.153158	0.903416
C	0.971205	-0.604015	1.778868
H	2.811570	-0.441084	-0.204292
H	2.381150	0.027335	-2.555205
C	-1.378878	-0.441091	2.220394
H	1.984986	-0.786955	2.108343
H	-2.211122	-0.507395	2.900933
C	-0.060269	-0.678373	2.688678
C	0.242285	-1.041126	4.131313
H	1.108937	-1.699137	4.113986
O	-0.079497	0.493678	-3.614514
C	0.993579	0.624688	-4.551858
H	1.561664	-0.303595	-4.634619

H	0.523290	0.845798	-5.505836
H	1.657864	1.441310	-4.269966
C	0.634155	0.183290	5.009238
O	-0.131647	1.180871	4.989877
C	-0.905014	-1.781000	4.836573
H	-0.574443	-2.130841	5.816077
H	-1.760760	-1.124069	4.988812
H	-1.232041	-2.636321	4.250377
O	1.669714	0.057663	5.720426
